# Inhibition of mammalian mtDNA transcription acts paradoxically to reverse diet-induced hepatosteatosis and obesity

**DOI:** 10.1038/s42255-024-01038-3

**Published:** 2024-04-30

**Authors:** Shan Jiang, Taolin Yuan, Florian A. Rosenberger, Arnaud Mourier, Nathalia R. V. Dragano, Laura S. Kremer, Diana Rubalcava-Gracia, Fynn M. Hansen, Melissa Borg, Mara Mennuni, Roberta Filograna, David Alsina, Jelena Misic, Camilla Koolmeister, Polyxeni Papadea, Martin Hrabe de Angelis, Lipeng Ren, Olov Andersson, Anke Unger, Tim Bergbrede, Raffaella Di Lucrezia, Rolf Wibom, Juleen R. Zierath, Anna Krook, Patrick Giavalisco, Matthias Mann, Nils-Göran Larsson

**Affiliations:** 1 Department of Medical Biochemistry and Biophysics, Karolinska Institutet, Stockholm, Sweden; 2 Department of Proteomics and Signal Transduction, Max-Planck Institute of Biochemistry, Martinsried, Germany; 3 University of Bordeaux, CNRS, Institut de Biochimie et Génétique Cellulaires (IGBC) UMR, Bordeaux, France; 4 Institute of Experimental Genetics - German Mouse Clinic, Helmholtz Zentrum, Munich, Germany; 5 German Center for Diabetes Research (DZD), Oberschleißheim-Neuherberg, Neuherberg, Germany; 6 Department of Physiology and Pharmacology, Section for Integrative Physiology, Karolinska Institutet, Stockholm, Sweden; 7 Chair of Experimental Genetics, TUM School of Life Sciences, Technische Universität München, Freising, Germany; 8 Department of Cell and Molecular Biology, Karolinska Institutet, Stockholm, Sweden; 9 Lead Discovery Center, Dortmund, Germany; 10 Centre for Inherited Metabolic Diseases, Karolinska University Hospital, Stockholm, Sweden; 11 Department of Molecular Medicine and Surgery, Section for Integrative Physiology, Karolinska Institutet, Stockholm, Sweden; 12 Metabolomics Core Facility, Max Planck Institute for Biology of Ageing, Cologne, Germany

**Keywords:** Fat metabolism, Energy metabolism, Obesity, Metabolism

## Abstract

The oxidative phosphorylation system^1^ in mammalian mitochondria plays a key role in transducing energy from ingested nutrients^2^. Mitochondrial metabolism is dynamic and can be reprogrammed to support both catabolic and anabolic reactions, depending on physiological demands or disease states. Rewiring of mitochondrial metabolism is intricately linked to metabolic diseases and promotes tumour growth^3–5^. Here, we demonstrate that oral treatment with an inhibitor of mitochondrial transcription (IMT)^6^ shifts whole-animal metabolism towards fatty acid oxidation, which, in turn, leads to rapid normalization of body weight, reversal of hepatosteatosis and restoration of normal glucose tolerance in male mice on a high-fat diet. Paradoxically, the IMT treatment causes a severe reduction of oxidative phosphorylation capacity concomitant with marked upregulation of fatty acid oxidation in the liver, as determined by proteomics and metabolomics analyses. The IMT treatment leads to a marked reduction of complex I, the main dehydrogenase feeding electrons into the ubiquinone (Q) pool, whereas the levels of electron transfer flavoprotein dehydrogenase and other dehydrogenases connected to the Q pool are increased. This rewiring of metabolism caused by reduced mtDNA expression in the liver provides a principle for drug treatment of obesity and obesity-related pathology.

## Abstract (web summary)

Inhibitor of mitochondrial transcription treatment leads to reduced oxidative phosphorylation capacity but increases fatty acid oxidation in the liver, leading to protection from obesity and related pathology.

## Main

The first attempts to target mitochondria to treat obesity were reported in the 1930s when more than 100,000 individuals were treated with the uncoupler dinitrophenol (DNP)^7–9^. Although this treatment increased the metabolic rate and reduced obesity, serious side effects prevented DNP from becoming an established treatment^7,8^. Metformin provides an alternate way to inhibit oxidative phosphorylation (OXPHOS) and this mild complex I inhibitor is widely used as an anti-diabetic medication and also protects against cancer^10–15^. The possible connection between beneficial metabolic effects and anti-cancer activity of drugs targeting mitochondria prompted us to investigate whether inhibitor of mitochondrial transcription (IMT) treatment, which is known to impair tumour metabolism and growth in mouse models^6^, also may have beneficial metabolic effects. Treatment of tumour cell lines with IMT induces a dose-dependent impairment of OXPHOS and cellular metabolic starvation, with progressively reduced levels of a range of critical metabolites and eventually cell death^6^. Despite the drastic effects on metabolism in cancer cell lines and cancer xenografts, treatment of whole animals is well tolerated^6^. We therefore decided to test the hypothesis that IMT treatment aiming to moderately impair the OXPHOS capacity in whole animals may induce beneficial metabolic effects in healthy and metabolically challenged mice.

**Fig. 1:**
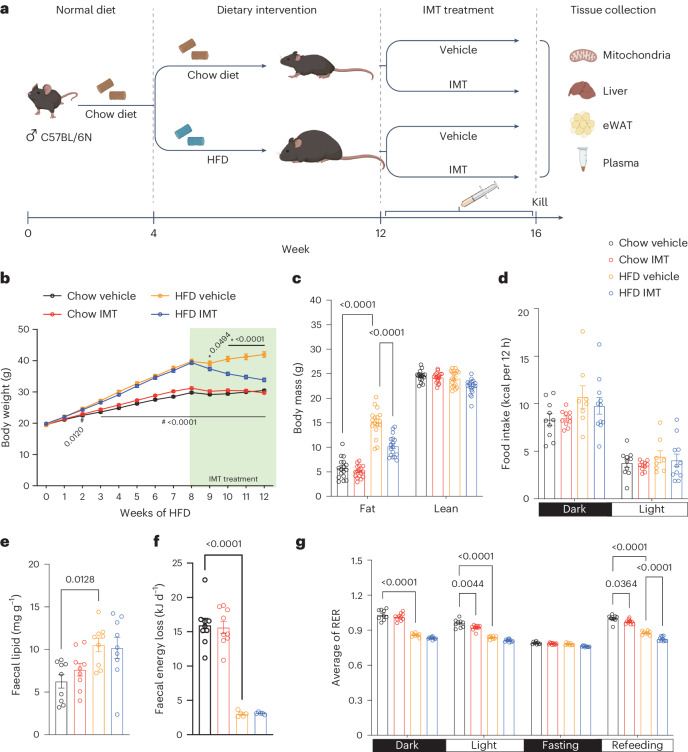
IMT treatment prevents diet-induced obesity and improves glucose homoeostasis. **a**, Experimental strategy for diet intervention and IMT treatment. Male 4-week-old C57BL/6N mice were randomly fed either a chow diet or HFD for 8 weeks. Thereafter, the diet was continued and mice were orally treated with IMT (30 mg kg^−1^) or vehicle for 4 weeks. Six independent cohorts of mice were used in this study; total mice *n* = 260. **b**, Body weight in mice on a chow diet or HFD treated with vehicle or IMT compound; *n* = 22 mice per group. Asterisk indicates a significant difference between HFD IMT and HFD vehicle. ^#^Indicates a significant difference between chow vehicle and HFD vehicle. *P* values are indicated. **c**, Body composition showing fat mass and lean mass after 4 weeks of IMT treatment; *n* = 17 mice per group. **d**,**g**, Measurement of whole-body metabolism during the fourth week of gavage treatment with vehicle or IMT compound using Oxymax/CLAMS. Food intake during the fourth day (**d**). The average RER over 42 h during the light and dark cycles (**g**). The RER in the four groups of mice. Chow vehicle, *n* = 10; chow IMT, *n* = 10; HFD vehicle, *n* = 8; and HFD IMT, *n* = 11 mice. **e**, Mouse faeces was collected for 4 days during the fourth week of IMT or vehicle treatment using the Single Mouse Metabolic Cage System. Faecal lipids were extracted using Folch’s method; *n* = 9 mice per group. **f**, Total faecal energy was analysed using bomb calorimetry; *n* = 9 mice per group. Data are presented as mean ± s.e.m. (**b**–**g**). Statistical significance was assessed by a two-way ANOVA with Tukey’s test for multiple comparisons. *P* values are indicated. Part of the image in **a** was created with BioRender.com. Source data

**Extended Data Fig. 1:**
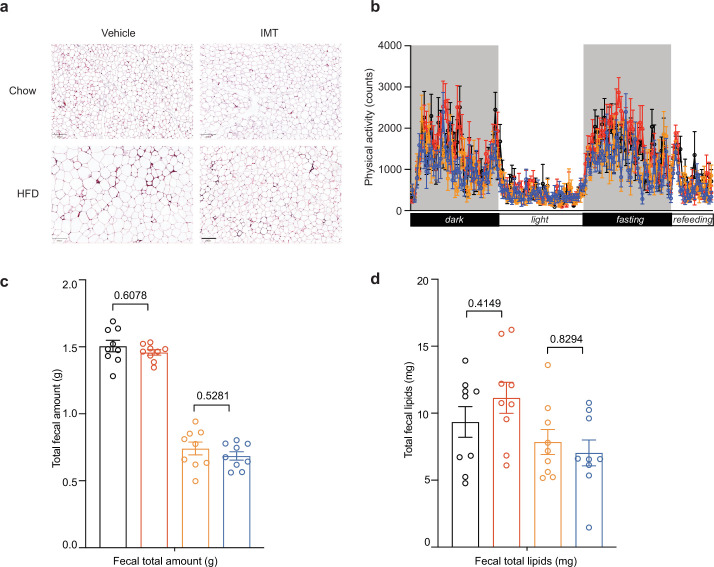
IMT treatment reduces adiposity without affecting physical activity and total faecal content. **a**, Representative images of H&E staining of eWAT. Scale bars, 200 µm. n = 5 mice per group. **b**, Measurement of whole-body metabolism during the fourth week of gavage treatment with vehicle or IMT compound by using the Oxymax/Comprehensive Lab Animal Monitoring System (CLAMS). The first three days were used to acclimate the animals to the CLAMS system, followed by measurements during the fourth day. Physical activity during day four and day five. Chow vehicle n = 10, Chow IMT n = 10, HFD Vehicle n = 8, and HFD IMT n = 11 mice. Total faecal amount (**c**) and total faecal lipid content (**d**) in mice. **c** and **d**, Mouse faeces was collected using the Single Mouse Metabolic Cage System. Faecal lipids were extracted using Folch’s method. n = 9 mice per group. All data are presented as mean ± SEM. Statistical significance was assessed by a two-way ANOVA with Tukey’s test for multiple comparisons. P values are shown in the figure. Source data

Male C57BL/6N mice at the age of 4 weeks were randomly chosen to be fed a chow diet or high-fat diet (HFD) for 8 weeks. Thereafter, the two groups were subdivided for oral treatment (gavage) with either IMT (LDC4857, 30 mg kg^−1^) or vehicle for 4 weeks while continuing the respective diets (Fig. 1a). The IMT compound used in this study was developed within an optimization programme based on the structurally closely related IMT1B compound. IMT treatment of mice on HFD causes a rapid marked reduction of body weight after 1 week, with a cumulative weight loss of ~7 g after 4 weeks (Fig. 1b). Measurements of body composition with non-invasive magnetic resonance imaging (EchoMRI-100) after 4 weeks of IMT treatment showed markedly reduced fat mass without any change of lean mass (Fig. 1c). Haematoxylin and eosin (H&E) staining of tissue sections of epididymal white adipose tissue (eWAT) showed that HFD results in large lipid-filled adipocytes and that IMT treatment leads to a drastic decrease in adipocyte size (Extended Data Fig. 1a).

We next assessed whole-body energy homoeostasis in mice on chow diet or HFD treated with vehicle or the IMT compound using the Oxymax/Comprehensive Lab Animal Monitoring System (CLAMS). The four groups of mice were subjected to five continuous days of CLAMS analysis during the fourth week of gavage treatment with vehicle or IMT compound. The first 3 days were used to acclimate the animals to the CLAMS system, followed by measurements during the fourth day. Day five included a 12-h period of fasting followed by 6 h of refeeding. Notably, IMT treatment did not alter food intake (Fig. 1d) or physical activity (Extended Data Fig. 1b). We found that IMT treatment did not increase the lipid content in faeces (Fig. 1e) or the total diurnal lipid excretion in faeces (Extended Data Fig. 1c,d). We performed bomb calorimetry and found a higher energy content in faeces in mice on a chow diet in comparison with mice on HFD, consistent with results from other studies^16,17^. IMT treatment did not additionally alter the total energy content in faeces (Fig. 1f). These analyses of faeces thus exclude that drug-induced malabsorption explains the weight loss.

**Extended Data Fig. 2:**
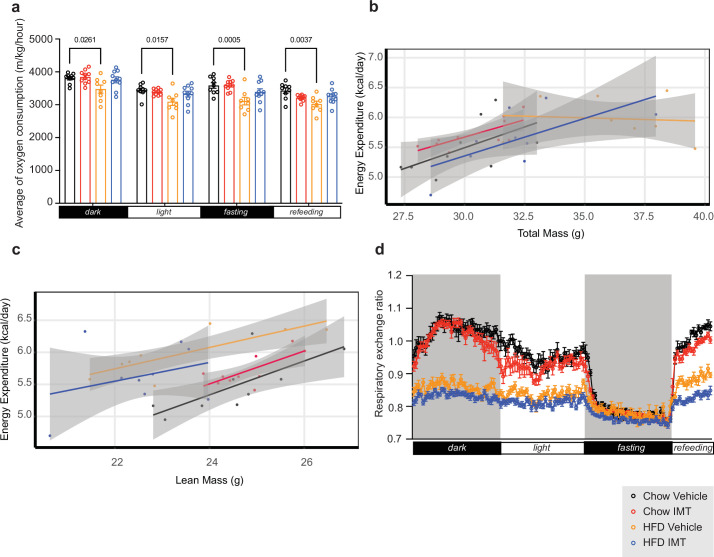
Measurement of whole-body metabolism in mice. Measurement of whole-body metabolism during the fourth week of gavage treatment with vehicle or IMT using CLAMS. **a**, The average oxygen consumption rate during day four and day five. Regression plot of energy expenditure versus total mass (**b**), or lean mass (**c**). **d**, Respiratory exchange ratio during day four and day five. **a**-**d**, Chow vehicle n = 9, Chow IMT n = 10, HFD Vehicle n = 8, and HFD IMT n = 10 mice. All data are presented as mean ± SEM. Statistical significance was assessed by a two-way ANOVA with Tukey’s test for multiple comparisons. P values are shown in the figure. Source data

IMT-treated mice on HFD showed enhanced oxygen consumption during both the light and dark cycle (Extended Data Fig. 2a). Regression-based analysis of covariance (ANCOVA)^18,19^ with either total mass or lean mass as a covariate did not clearly link increased energy expenditure to IMT treatment (Extended Data Fig. 2b,c). Although these results indicate that IMT treatment may not exert its effect through increasing energy expenditure, subtle differences in energy expenditure can be hard to detect by indirect calorimetry despite having a profound long-term impact on body weight^18,20^. We therefore proceeded to assess the respiratory exchange ratio (RER) as this parameter needs no normalization to body weight or body composition. Mice on a standard chow diet had a RER of ~0.9–1.1, whereas it was decreased to ~0.8 on HFD (Fig. 1g and Extended Data Fig. 2d), as expected. Upon refeeding after fasting, IMT treatment resulted in a lower RER in comparison with vehicle treatment, regardless of the diet (Fig. 1g and Extended Data Fig. 2d), consistent with drug-induced activation of fat metabolism. These data provide evidence that IMT treatment reverses HFD-induced obesity by promoting metabolism of fat at the organismal level.

**Extended Data Fig. 3:**
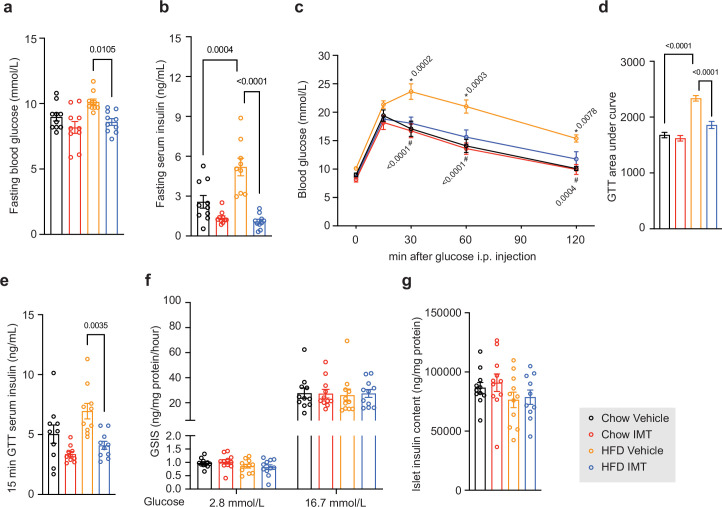
IMT improves glucose homoeostasis and does not impair islet insulin secretion. **a**, **b**, Fasting blood glucose (**a**) and fasting serum insulin (**b**) levels in mice after four weeks of IMT treatment. n = 10 mice per group. **c, d**, Blood glucose levels (**c**) and the area under the curve (AUC, **d**) during intraperitoneal glucose tolerance tests (ipGTT) with 2 g/kg glucose in mice after four weeks of vehicle or IMT treatment. n = 10 mice per group. Data are presented as mean ± SEM. Statistical significance was assessed by a two-way ANOVA with Tukey’s test for multiple comparisons. ∗ indicates a significant difference between HFD IMT and HFD Vehicle; ^#^ indicates a significant difference between Chow Vehicle and HFD Vehicle. P values are shown in the figure. **e**, Serum insulin levels at the 15-min of ipGTT. n = 10 mice per group. **f**, Ex vivo glucose-stimulated insulin secretion assays performed on isolated pancreatic islets. Glucose (2.8 mM and 16.7 mM) was added to the medium to recapitulate basal and glucose-stimulated insulin secretion conditions. Three independent GSIS assays were performed with three replicates per group. **g**, Islet insulin content. Three independent GSIS assays were performed with three replicates per group. All data are presented as mean ± SEM. Statistical significance was assessed by a two-way ANOVA with Tukey’s test for multiple comparisons. P values are shown in the figure. Source data

We found normal fasting blood glucose levels accompanied by markedly increased fasting serum insulin levels (Extended Data Fig. 3a,b) and pathological intraperitoneal glucose tolerance tests (ipGTT; Extended Data Fig. 3c–e) with an increased peak concentration of serum insulin (Extended Data Fig. 3e) in mice on HFD, consistent with a pre-diabetic state and insulin resistance. Glucose homoeostasis was markedly improved when mice on HFD were treated with an IMT compound for 4 weeks; the fasting blood glucose was reduced (Extended Data Fig. 3a), serum insulin levels were decreased (Extended Data Fig. 3b) and the ipGTT responses were normalized (Extended Data Fig. 3c–e). IMT treatment leads to reduced circulating insulin levels (Extended Data Fig. 3b), but ex vivo glucose-stimulated insulin secretion (GSIS) assays showed that IMT treatment did not impair insulin secretion or insulin biosynthesis in isolated pancreatic islets (Extended Data Fig. 3f,g). The reduced circulating insulin levels and normalized glucose homoeostasis in IMT-treated mice on HFD are, thus, probably explained by increased insulin sensitivity.

**Fig. 2:**
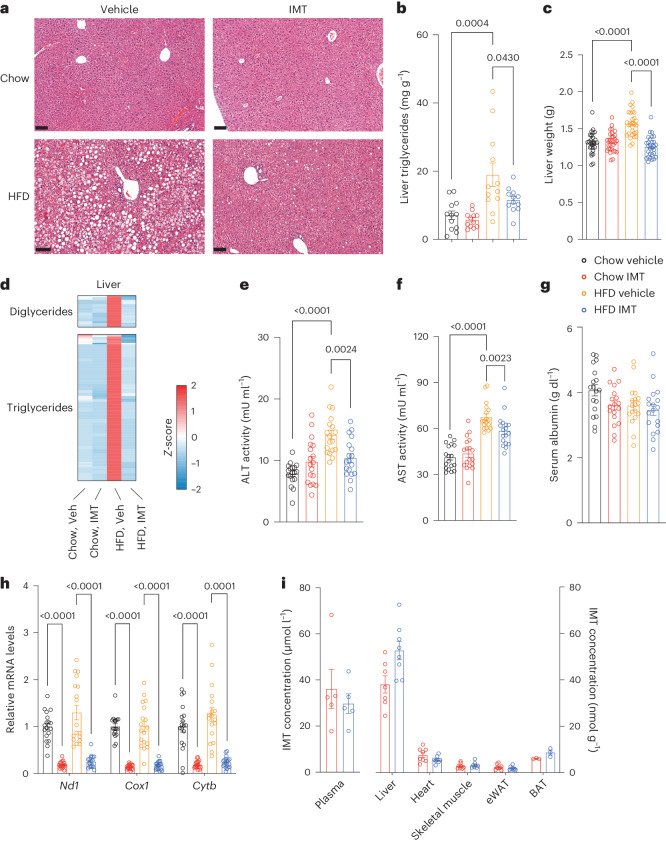
IMT treatment reverses hepatosteatosis. **a**, Representative images of H&E staining showing liver structure and morphology in mice on a chow diet or HFD treated with either vehicle or IMT compound. Scale bars, 100 µm. *n* = 5 mice per group. **b**, Quantitative measurement of triglycerides in mouse liver after 4 weeks of IMT treatment; *n* = 12 mice per group. **c**, Liver weight in mice treated with vehicle or IMT for 4 weeks; *n* = 30 mice per group. **d**, The levels of diglycerides and triglycerides in mouse liver after 4 weeks of IMT treatment. Chow vehicle, chow IMT and HFD vehicle, *n* = 8 mice per group; HFD IMT, *n* = 7 mice. Veh, vehicle. **e**–**g**, Serum alanine aminotransferase (ALT) activity (**e**) aspartate aminotransferase (AST) activity (**f**) and albumin levels (**g**) measured in mice after 4 weeks of vehicle or IMT treatment; *n* = 18 mice per group. **h**, Mitochondrial transcript levels in the liver after 4 weeks of IMT treatment; *n* = 12 mice per group. **i**, IMT concentration in plasma and mouse tissues. Plasma, *n* = 5 mice per group; liver chow IMT, *n* = 7 mice; HFD IMT, *n* = 8 mice; heart, skeletal muscle, eWAT, *n* = 8 mice per group; BAT, *n* = 3 mice per group. Data are presented as mean ± s.e.m. (**b**,**c**,**e**–**i**). Statistical significance was assessed by a two-way ANOVA with Tukey’s test for multiple comparisons (**b**,**c**,**e**,**g**,**i**) and a Mann–Whitney *U*-test (**f**,**h**). *P* values are indicated. Source data

**Extended Data Fig. 4:**
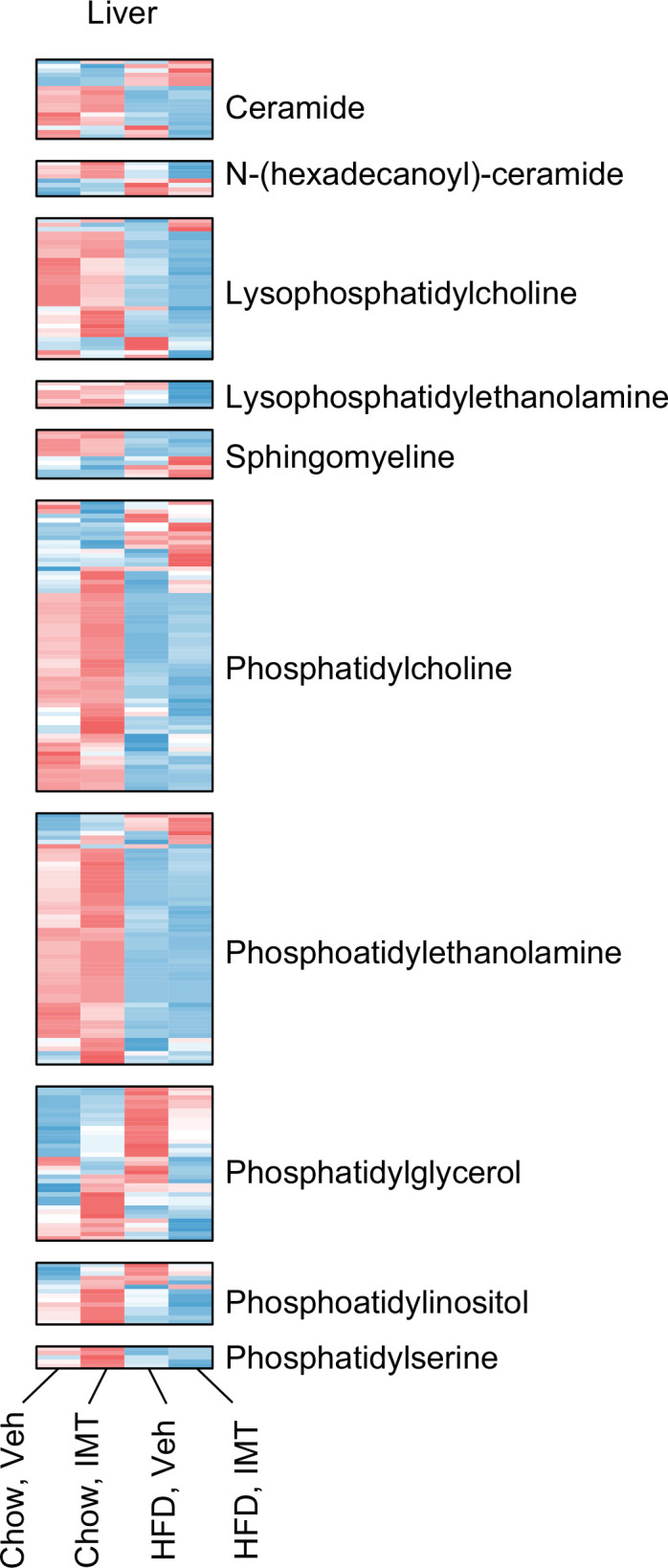
IMT treatment does not change levels of phospholipids and sphingolipids. Levels of phospholipids and sphingolipids in mouse liver after four weeks of vehicle or IMT treatment. n = 8 mice per group. Source data

We observed a large macrovesicular steatosis in the liver of mice on HFD (Fig. 2a). Notably, IMT treatment markedly reduced hepatosteatosis (Fig. 2a), leading to a decreased lipid content in the liver (Fig. 2b) and reduced liver weight (Fig. 2c). We performed lipidomics and found a large accumulation of diglycerides and triglycerides in the liver of mice on HFD, which was reversed by 4 weeks of IMT treatment (Fig. 2d). In contrast, the phospholipid and sphingolipid levels in the liver were mainly affected by the diet and not markedly impacted by IMT treatment (Extended Data Fig. 4). IMT treatment of mice on HFD was accompanied by an improvement of the liver function, as demonstrated by decreased aminotransferase activities in the serum (Fig. 2e,f). The serum albumin levels were normal in all investigated groups (Fig. 2g). Taken together these data show that IMT treatment can reverse diet-induced hepatosteatosis and normalize liver function.

**Extended Data Fig. 5:**
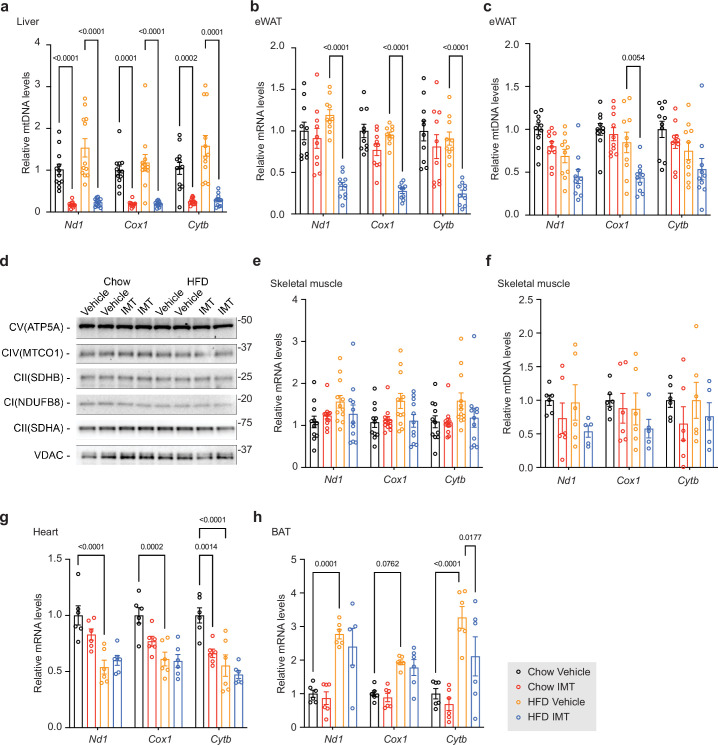
Levels of mitochondrial transcripts and mtDNA in different tissues of IMT-treated mice. **a-f**, Levels of representative mitochondrial transcripts and mtDNA were measured in tissues of mice on chow diet or HFD treated with vehicle or IMT compound for four weeks. Levels of mtDNA in liver after four weeks of IMT treatment. n = 12 mice per group (**a**). The mtDNA transcript (**b**) and mtDNA (**c**) levels in eWAT. n = 10 mice per group. **d**, Representative western blot analyses of OXPHOS protein levels in eWAT after four weeks of vehicle or IMT treatment. Subunits of complex I (NDUFB8), complex II (SDHA and SDHB), complex IV (MTCOX1), and complex V (ATP5A) were analysed. VDAC was used as loading control. A representative image of n = 3 independent experiments is shown. The mtDNA transcript, n = 10 mice per group (**e**) and mtDNA, n = 6 mice per group (**f**) levels in skeletal muscle. The mtDNA transcript levels in heart (**g**) and brown adipose tissue (BAT, **h**). n = 6 mice per group. All data are presented as mean ± SEM. **a**, **e**, Statistical significance was assessed by the Mann–Whitney U-test. **b**, **c**, **f**-**h**, Statistical significance was assessed by a two-way ANOVA with Tukey’s test for multiple comparisons. P values are shown in the figure. Source data

IMT treatment resulted in marked reduction in levels of mtDNA-encoded transcripts (Fig. 2h) and mtDNA (Extended Data Fig. 5a) in the livers of mice on a chow diet or HFD. The decrease of mtDNA is probably due to a decreased formation of the RNA primers needed for initiation of mtDNA replication, because IMT inhibits POLRMT, which is not only necessary for gene expression but also serves as the primase for mammalian mtDNA replication^21–23^. Treatment with IMT resulted in a moderate decrease of mtDNA-encoded transcripts and mtDNA levels in eWAT (Extended Data Fig. 5b,c), but there was no decrease in levels of OXPHOS subunits (Extended Data Fig. 5d). No significant changes in the levels of mtDNA-encoded transcripts or mtDNA levels were observed in skeletal muscle after IMT treatment (Extended Data Fig. 5e,f). No significant changes in levels of mtDNA-encoded transcripts were observed in the heart (Extended Data Fig. 5g) and brown adipose tissue (BAT) (Extended Data Fig. 5h) after IMT treatment.

To gain further insights into the differences in inhibition of mtDNA transcription between tissues, we measured IMT concentrations 24 h after the last dose in mice treated with IMT for 4 weeks (Fig. 2i). The IMT concentrations were much higher in the plasma and liver than in the heart, skeletal muscle, eWAT and BAT (Fig. 2i), which shows that the IMT compound is preferentially accumulated in the liver due to the oral route of administration and the first-passage effect. The skewed tissue distribution of IMT, thus, explains the preferential strong inhibitory effect on mtDNA transcription in liver.

**Extended Data Fig. 6:**
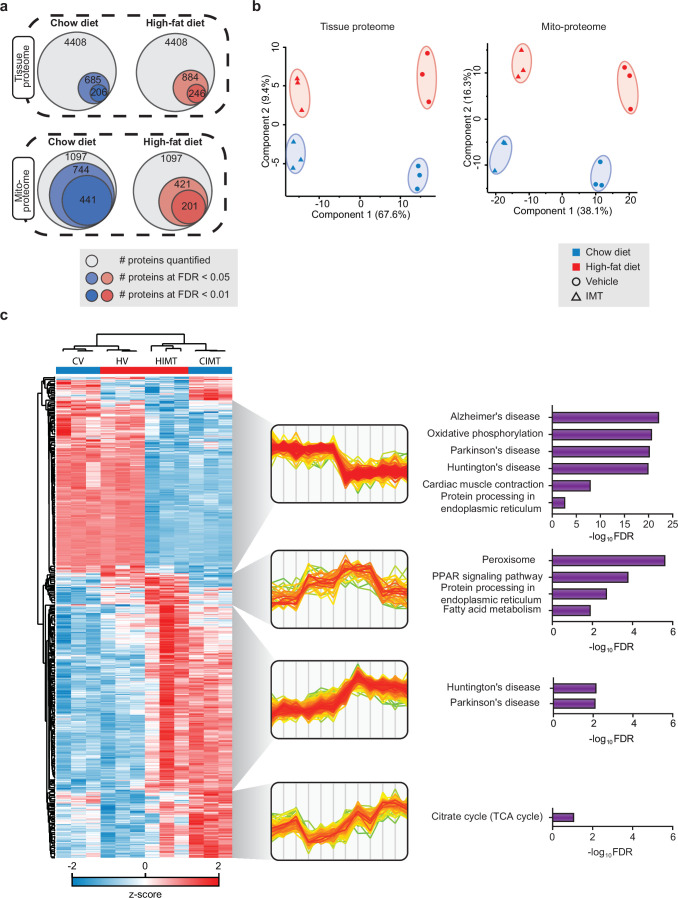
Analysis of the total and mitochondrial proteome. **a**, Venn diagram showing number of quantified and significantly changed proteins at a given FDR cutoff. The liver tissue proteome and the proteome of isolated liver mitochondria (Mitoproteome) are shown. **b**, Principal-component analyses (PCA) of the liver tissue and liver mitochondrial proteomes. **c**, Hierarchical clustering analysis of the total proteome (ANOVA-significant proteins at FDR < 5%; left) with biological replicates as individual lanes (CV: control vehicle; HV: HFD Vehicle; HIMT: HFD IMT-treated, CIMT: control IMT-treated), z-score normalised fold changes in indicated clusters (middle) with the same sample order as in the heatmap, and significant KEGG terms in each cluster (Fisher exact test at FDR < 5%; right). Source data

We used label‐free quantitative proteomics to identify differentially expressed proteins in the homogenates from liver tissue or ultra-purified liver mitochondria. In total, 4,408 proteins were identified in the liver tissue proteome of mice on a chow diet or HFD, and IMT treatment caused a significant change in the levels of ~15–20% of these proteins (false discovery rate (FDR) < 0.05, Extended Data Fig. 6a). A high proportion (68.7% at FDR < 0.05) of the proteins whose levels changed significantly after IMT treatment were classified as mitochondrial proteins, according to MitoCarta 3.0 (ref. ^24^). We performed principal-component analyses (PCA) and found that changes in both the total and mitochondrial liver proteome were mainly determined by the IMT treatment (Extended Data Fig. 6b).

**Extended Data Fig. 7:**
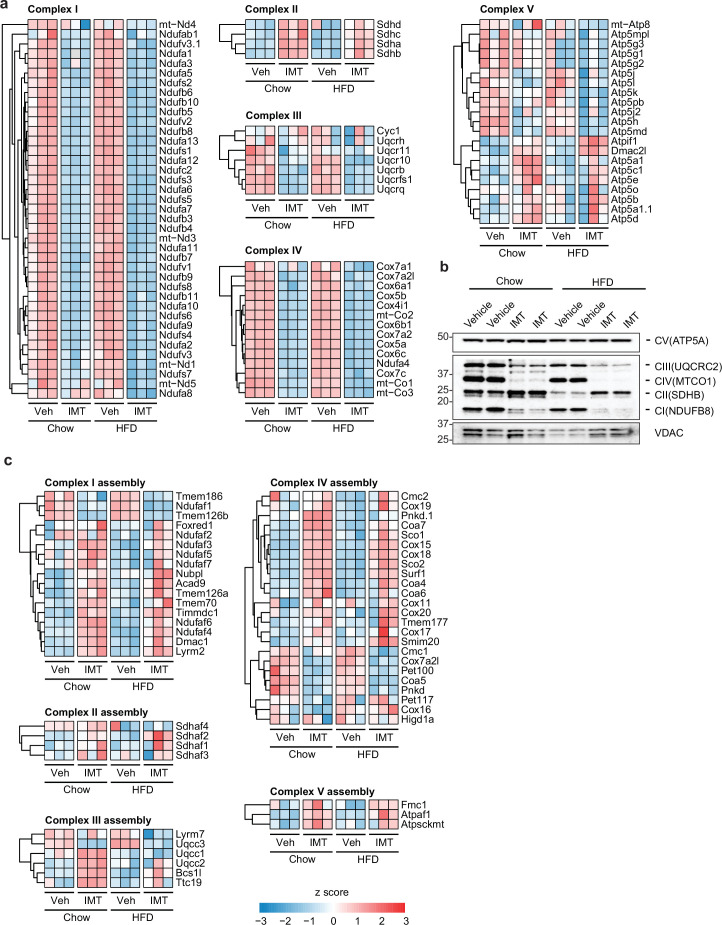
IMT treatment rewires OXPHOS. **a**, Heatmaps illustrating the protein density of subunits of OXPHOS complexes in mouse liver after four weeks of vehicle or IMT treatment. n = 3 mice per group. **b**, Representative western blot analyses of OXPHOS protein levels in liver mitochondria after four weeks of vehicle or IMT treatment. Subunits of complex I (NDUFB8), complex II (SDHB), complex III (UQCRC2), complex IV (MTCOX1), and complex V (ATP5A) were analysed. VDAC was used as loading control. A representative image of n = 3 independent experiments is shown. **c**, Heatmaps depicting the protein density of different mitochondrial OXPHOS assembly factors in mouse liver after four weeks of vehicle or IMT treatment. n = 3 mice per group. Source data

**Extended Data Fig. 8:**
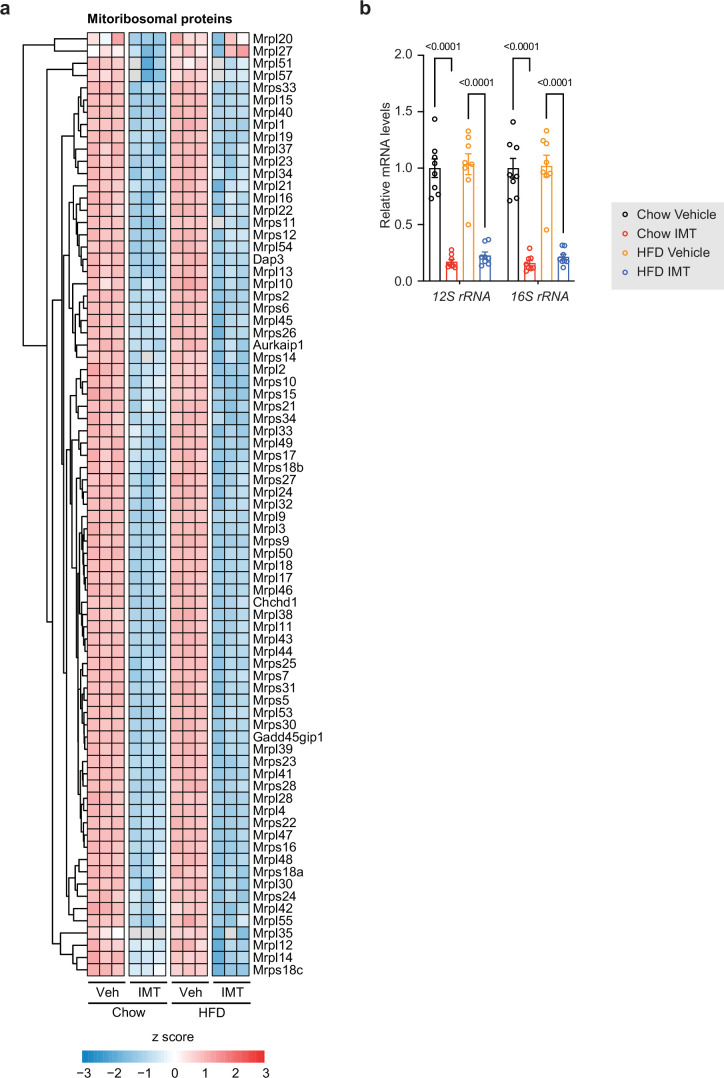
IMT decreases mitoribosomal proteins and rRNAs. **a**, Heatmaps depicting the protein density of mitoribosomal proteins in mouse liver after four weeks of vehicle or IMT treatment. n = 3 mice per group. **b**, The mtDNA-encoded 12S and 16S rRNA transcripts in mouse liver after four weeks of vehicle or IMT treatment. n = 8 mice per group. Data are presented as mean ± SEM. Statistical significance was assessed by a two-way ANOVA with Tukey’s test for multiple comparisons. P values are shown in the figure. Source data

A detailed inspection of the OXPHOS subunits in the mitochondrial proteome showed that IMT treatment significantly decreased the levels of subunits of complex I, III, IV and the membrane portion (F_0_) of complex V, whereas subunits of the matrix portion (F_1_) of complex V were less affected or even increased (Extended Data Fig. 6c and Extended Data Fig. 7a). In contrast, the levels of subunits of complex II (succinate dehydrogenase) were increased (Extended Data Fig. 7a), consistent with the lack of mtDNA-encoded subunits in this complex. Western blot analyses confirmed the reduction in the levels of OXPHOS complexes containing mtDNA-encoded subunits (Extended Data Fig. 7b). We also observed an increase in the levels of many OXPHOS complex assembly factors (Extended Data Fig. 7c). Levels of most of the mitochondrial ribosomal proteins were drastically decreased (Extended Data Fig. 8a) because IMT treatment decreases the 12S and 16S rRNA levels (Extended Data Fig. 8b) necessary for assembly of the mitochondrial ribosome.

**Extended Data Fig. 9:**
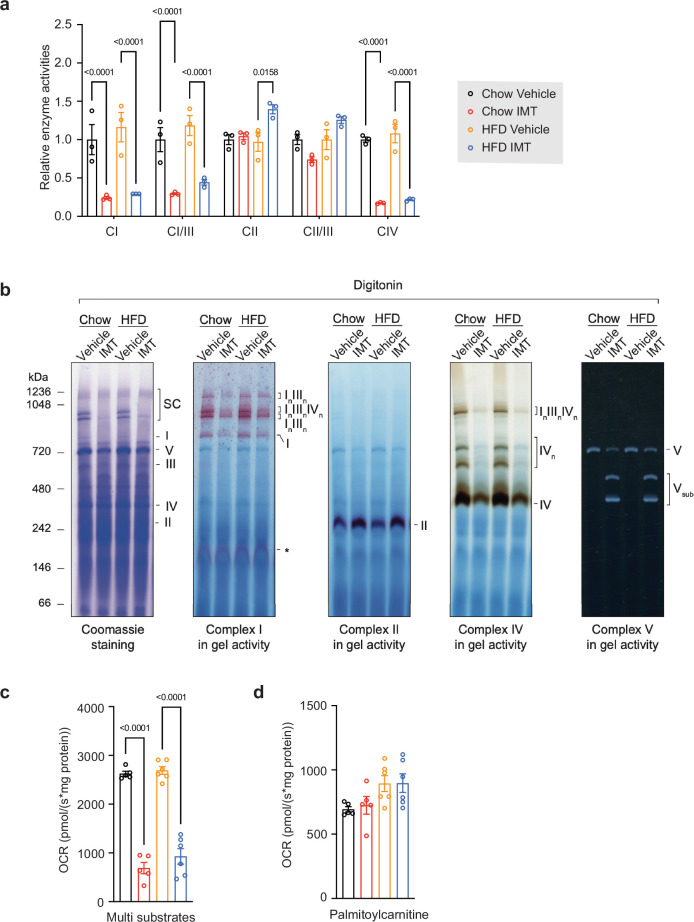
Characterization of liver OXPHOS function. **a**, Respiratory chain complex activities normalized to citrate synthase activity in liver mitochondria after four weeks of vehicle or IMT treatment. n = 3 mice per group. The analysed enzyme activities are NADH coenzyme Q reductase (complex I, CI), NADH cytochrome c reductase (complex I/III, CI/III), succinate dehydrogenase (complex II, CII), and cytochrome c oxidase (complex IV, CIV). **b**, In-gel activities of OXPHOS complexes resolved by BN-PAGE. Digitonin-solubilized liver mitochondria (60 μg) from mice on chow diet or HFD and subjected to vehicle or IMT treatment were resolved by native gel electrophoresis (BN-PAGE). Gels were stained with Coomassie or incubated with substrates for detecting the in-gel activity of the indicated OXPHOS complexes. Complex V subassembly intermediates containing F_1_ subunits (Vsub) are observed in the IMT-treated subunits. The image is representative of 3 biological replicates from each group. Oxygen consumption rate (OCR) of liver mitochondria in the state 3 with (**c**) multiple substrates (malate/palmitoylcarnitine, pyruvate/malate/glutamate, succinate and glycerophosphate), (**d**) malate/palmitoylcarnitine only. c and d, n = 5 mice/group. a, c, and d, data are presented as mean ± SEM. Statistical significance was assessed by a two-way ANOVA with Tukey’s test for multiple comparisons. P values are shown in the figure. Source data

We measured the activity of respiratory chain enzymes in liver mitochondria and found that IMT treatment caused a marked decrease in the activity of complex I, I/III and IV, whereas complex II and complex II/III activities were maintained (Extended Data Fig. 9a). Consistently, in-gel activities of OXPHOS complexes resolved by blue native polyacrylamide gel electrophoresis (BN-PAGE) showed a marked decrease in the levels and activities of assembled complex I and IV, and an increase in the level and activity of complex II in the liver mitochondria from IMT-treated mice irrespective of diet (Extended Data Fig. 9b). Furthermore, IMT treatment decreased the levels of fully assembled complex V and induced the appearance of a subassembly with ATPase activity (Extended Data Fig. 9b), consistent with proteomics analyses (Extended Data Fig. 7a) and previous observations of this sub-assembled complex in mouse models with reduced mtDNA gene expression^25,26^.

**Fig. 3:**
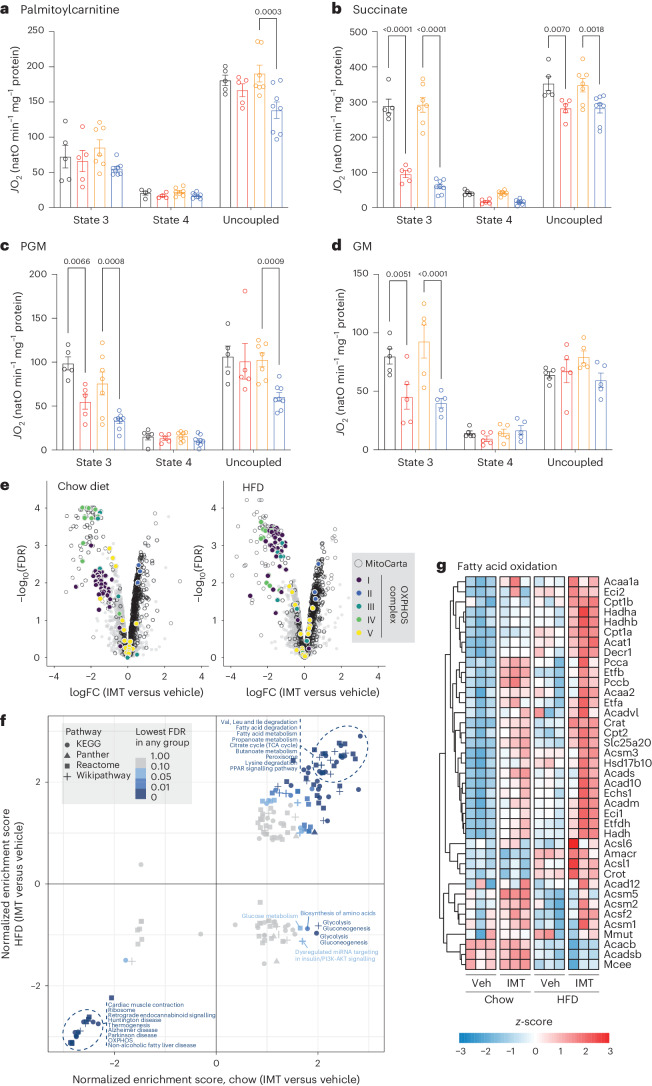
IMT treatment selectively maintains fatty acid respiration in liver mitochondria. **a**–**d**, Respiration of fresh liver mitochondria on malate/palmitoylcarnitine (**a**), succinate/rotenone (**b**), pyruvate/malate/glutamate (PGM) (**c**) and glutamate/malate (GM) (**d**) at state 3, state 4 and the uncoupled state. Chow vehicle and chow IMT groups, *n* = 5 mice per group; HFD vehicle, *n* = 7 mice; HFD IMT, *n* = 8 mice. Data are presented as mean ± s.e.m. Statistical significance was assessed by a two-way ANOVA with Tukey’s test for multiple comparisons. *P* values are indicated. *J*O_2_, oxygen consumption flux; natO, nanoatom oxygen. **e**, Volcano plot presenting all quantified proteins in mouse liver on a chow diet or HFD and subjected to vehicle or IMT treatment. The differentially expressed subunits of different OXPHOS complexes are highlighted in different colours. **f**, GSEA of total tissue and mitochondrial proteomes from the liver. **g**, Heatmaps illustrating the protein density of enzymes involved in fatty acid oxidation in mouse livers after 4 weeks of vehicle or IMT treatment; *n* = 3 mice per group (**e**–**g**). Source data

To further assess the impact of IMT treatment on bioenergetics, we performed high-resolution respiration experiments (Oroboros) on freshly isolated liver mitochondria from mice on a chow diet or HFD treated with vehicle or IMT. In the liver mitochondria from vehicle-treated mice, we observed additive effects on the oxygen consumption rate (OCR) when multiple substrates (palmitoylcarnitine, pyruvate + glutamate + malate, succinate and glycerol-1-phosphate) were sequentially added, but this additive effect was substantially reduced after IMT treatment (Extended Data Fig. 9c). In contrast, the oxidation of palmitoylcarnitine as a single substrate was similar in mouse liver mitochondria from animals treated with vehicle or IMT (Extended Data Fig. 9d). To assess OXPHOS function in more detail, respiration was measured using different combinations of substrates fuelling complex I (pyruvate, glutamate and malate), complex II (succinate and rotenone) and β-oxidation (palmitoylcarnitine) under phosphorylating (state 3), non-phosphorylating (pseudo-state 4 with oligomycin) and uncoupled states. In agreement with the first set of experiments (Extended Data Fig. 9d), we found maintained state 3 fatty acid oxidation in IMT-treated mice (Fig. 3a). Of note, despite the substantially impaired enzyme activity of complex I and IV (Extended Data Fig. 9a), the maximal uncoupled respiration was only mildly affected (Fig. 3b–d). In contrast, respiration assessed under phosphorylating conditions was severely impaired by the IMT treatment, suggesting that the phosphorylating respiration is highly controlled and impacted by ATP synthase deficiency (Fig. 3b–d). The IMT-induced ATP synthase deficiency selectively impaired complex I- and complex II-driven phosphorylating respiration (Fig. 3b–d), reducing the OCR to a similar level as β-oxidation-driven respiration (Fig. 3a). To summarize, the bioenergetic characterization of liver mitochondria from IMT-treated mice showed maintained fatty acid oxidation capacity despite markedly reduced OXPHOS capacity.

To further analyse the proteomics dataset, we used volcano plots and highlighted differentially expressed OXPHOS subunits in liver protein extracts from IMT-treated mice (Fig. 3e). The majority of subunits of complexes I, III and IV were downregulated, complex II subunits were upregulated, subunits of the F_1_ portion of complex V were increased, and subunits if the F_0_ portion of complex V were decreased (Fig. 3e), consistent with results from BN-PAGE (Extended Data Fig. 9b) and proteomics heatmaps (Extended Data Fig. 7a). To identify pathways influenced by IMT treatment, we performed gene set enrichment analysis (GSEA) of the total liver tissue proteome and found that enzymes involved in fatty acid metabolism/degradation were markedly enriched in the liver of IMT-treated mice, regardless of diet (Fig. 3f). The GSEA findings indicate that IMT rewires liver metabolism to favour fatty acid degradation, as documented by the increased levels of several key fatty acid oxidation enzymes, for example, the heterodimeric electron transfer flavoprotein subunits (ETFA and ETFB), electron transfer flavoprotein dehydrogenase (ETF-DH) and the carnitine acyltransferases (CPT1a and CPT2) (Fig. 3g).

**Extended Data Fig. 10:**
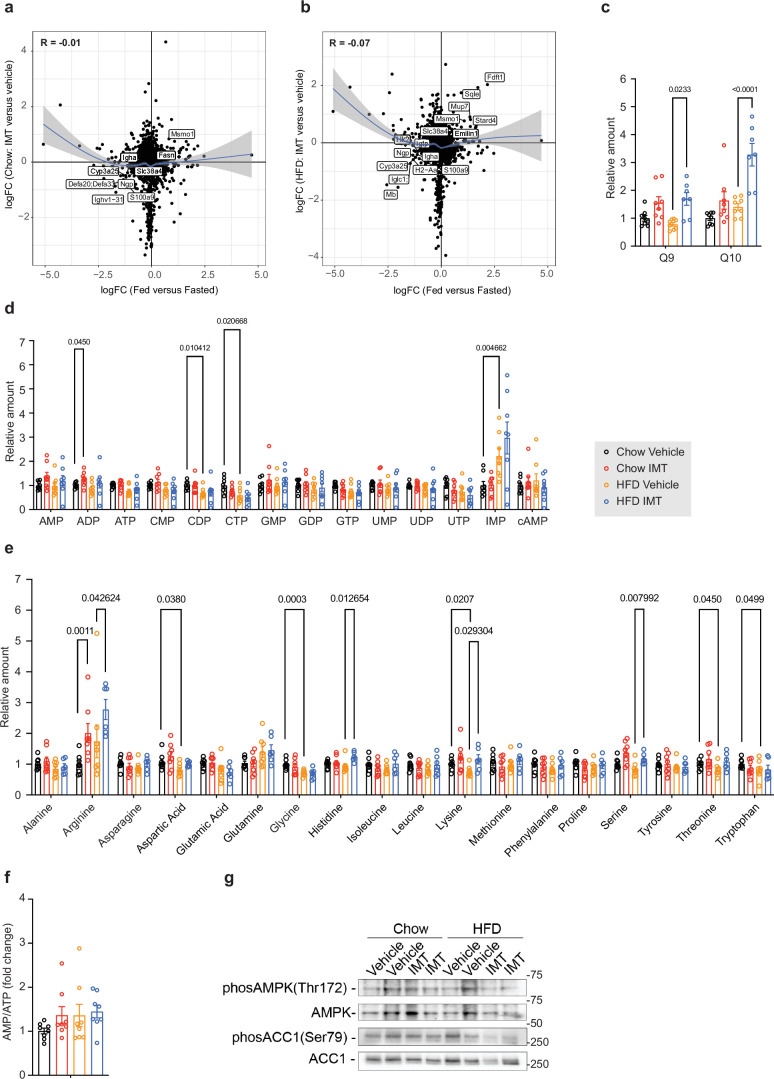
Analysis of the liver metabolites and fasting mouse proteomics correlation. **a, b**, Liver proteomics correlation between IMT/vehicle and the fed/fasted mice on chow diet (**a**) or HFD (**b**). n = 3 mice per group. **c**, Fold change in the levels of quinones (Q9 and Q10) in mouse liver after four weeks of vehicle or IMT treatment. Chow Vehicle, Chow IMT and HFD Vehicle n = 8 mice per group, HFD IMT n = 7 mice per group. **d**, Fold change in nucleotide levels in mouse liver after four weeks of vehicle or IMT treatment. n = 8 mice per group. **e**, Fold change in amino acid levels in mouse liver after four weeks of vehicle or IMT treatment. Chow Vehicle, Chow IMT and HFD Vehicle n = 8 mice per group, HFD IMT n = 6 mice. **f**, The ratio of AMP to ATP in mouse liver after four weeks of vehicle or IMT treatment. n = 8 mice per group. **c**-**f**, All data are presented as mean ± SEM. **c**, Statistical significance was assessed by a two-way ANOVA with Tukey’s test for multiple comparisons. **d-f**, Statistical significance was assessed by the Mann–Whitney U-test. P values are shown in the figure. **g**, Western blot analyses of the levels of AMPK phosphorylated at T172 (pAMPK(T172)) and ACC1 phosphorylated at (pACC1(S79)). A representative image of n = 2 independent experiments is shown. Source data

We proceeded to investigate whether fasting can induce changes in protein expression that are similar to those seen after IMT treatment. Age-matched C57BL/6N mice were fasted for 16 h and total liver protein extracts were used for label-free proteomics analyses (Extended Data Fig. 10a,b). We found nearly no correlation of liver protein expression between fasting and IMT-treated mice under chow diet (*R* = −0.01) or HFD (*R* = −0.07). Thus, the reversal of obesity, depending on IMT-induced activation of fatty acid oxidation in liver, does not mimic induction of a fasting-like response.

**Fig. 4:**
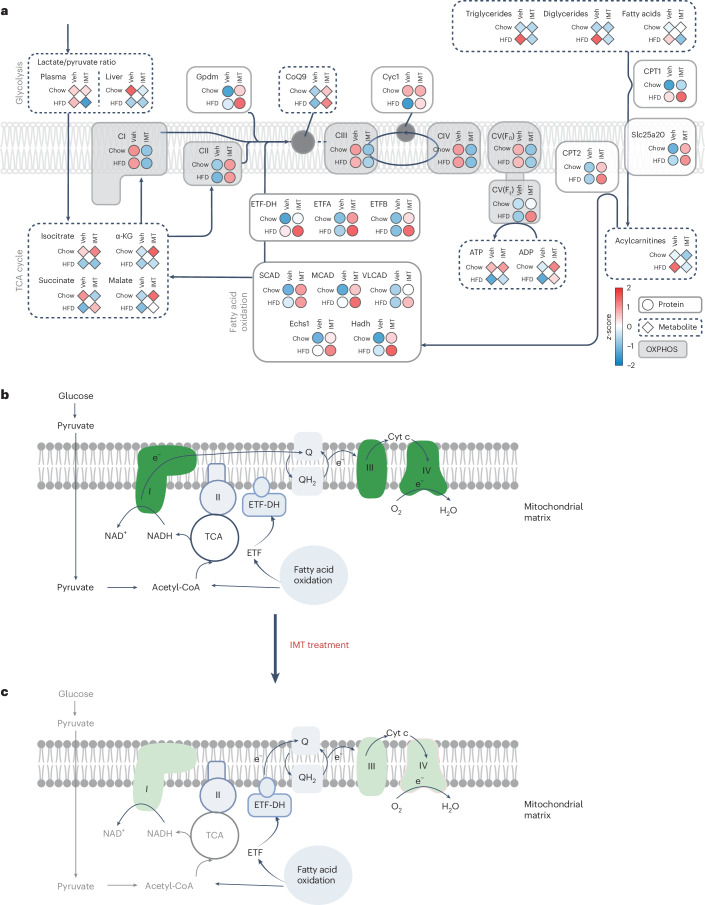
IMT treatment rewires liver metabolism. **a**, An integrated view of the changes of metabolite and protein levels in liver of mice on a chow diet or HFD and subjected to vehicle or IMT treatment. The protein levels are represented by circles and the metabolite levels are represented by diamonds. **b**, Substrate oxidation and electron transfer pathways to Q under normal conditions. Glucose is metabolized and produces pyruvate. Pyruvate is imported to the mitochondria and is converted to acetyl-CoA, which enters the tricarboxylic acid (TCA) cycle and generates NADH. Complex I oxidizes NADH and functions as the primary entry point for electrons into the Q pool. Electrons, thereafter, flow through complex III, then through cytochrome c and finally reach complex IV where they reduce molecular oxygen to water. **c**, IMT impairs the OXPHOS capacity and rewires the pathways for electron transfer to Q. IMT reduces the activities of complex I, III and IV. As a consequence, the capacity of complex I to oxidize NADH is reduced and the electron transfer through electron-transfer flavoprotein dehydrogenase (ETF-DH) to the Q pool is increased. IMT treatment, thus, impairs the OXPHOS system leading to a rewiring of liver metabolism that decreases OXPHOS capacity and maintains fatty acid oxidation. Images in **b** and **c** were created with BioRender.com. CI, complex I; CII, complex II; CIII, complex III; CIV, complex IV; CV, complex V; α-KG, α-ketoglutarate; Gpdm, mitochondrial glycerol phosphate dehydrogenase; CoQ9, ubiquinone biosynthesis protein COQ9, mitochondrial; SCAD, short chain acyl-CoA dehydrogenase; MCAD, medium chain acyl-CoA dehydrogenase; VLCAD, very long chain acyl-CoA dehydrogenase.

Next, we performed metabolomics to identify metabolites in the extracts from liver tissue after 4 weeks of IMT treatment in mice on a chow diet or HFD. We found that the levels of quinones (Q9 and Q10) were normal or increased (Extended Data Fig. 10c). When human cancer cell lines are treated with IMT1B, there is a time-dependent, marked decrease of triphosphate nucleotides (accompanied by an increase in mono- and diphosphate nucleotides) and a decrease in amino acids, which leads to a cellular energy crisis, activation of AMPK and cell death^6^. In contrast, metabolite analyses in the livers of IMT-treated mice on a chow diet or HFD showed normal levels of key mono-, di- and triphosphate nucleotides (Extended Data Fig. 10d) and amino acids (Extended Data Fig. 10e), consistent with the observation that liver function is not impaired by IMT treatment (Fig. 2e–g). The calculated ratio of AMP to ATP was not changed by IMT treatment (Extended Data Fig. 10f) and consistently, we found no difference in the phosphorylation of AMPK and the downstream acetyl CoA carboxylase (ACC) enzyme in the liver (Extended Data Fig. 10g). We proceeded with a comparison of proteomics and metabolomics data and found an overall pattern consistent with the activation of fatty acid oxidation in the liver of IMT-treated mice on HFD (Fig. 4a).

We traditionally think of the mammalian respiratory chain as consisting of complexes I–IV, where complex I and II both are dehydrogenases that directly deliver electrons to the Q pool^1^ (Fig. 4b); however, there are at least four additional dehydrogenases that are connected to the matrix- or intermembrane-space side of the inner mitochondrial membrane^27^. The OXPHOS system thus integrates many metabolic pathways through several different dehydrogenases that directly deliver electrons to the Q pool for subsequent electron translocation by complex III and IV, leading to the final reduction of molecular oxygen to water. Although the biogenesis of complex I is critically dependent on mtDNA expression^26,28^, the other dehydrogenases that directly deliver electrons to the Q pool are exclusively nucleus-encoded and are therefore not directly impacted by IMT treatment. The final step of fatty acid oxidation depends on the ETF dehydrogenase that directly delivers electrons to the Q pool for transfer to complex III and IV. Complex I is the main dehydrogenase in the OXPHOS system and it is possible that a reduction of its levels and/or activity allows better access for ETF-DH to directly deliver electrons to the Q pool (Fig. 4b,c). This type of competition between different dehydrogenases for electron delivery to the Q pool has been documented in budding yeast^29^ and recently between complex I and II in mammals^30^ and may also occur for other Q-oxidoreductases fuelling the respiratory chain.

We present a range of findings showing that IMT treatment has beneficial metabolic effects, causing (1) decreased weight (fat content) of mice on HFD (Fig. 1b,c and Extended Data Fig. 1a) without affecting food intake (Fig. 1d), physical activity (Extended Data Fig. 1b) or intestinal nutrient uptake (Fig. 1e,f and Extended Data Fig. 1c,d); (2) decreased RER consistent with a shift towards fatty acid oxidation at the organismal level (Fig. 1g and Extended Data Fig. 2d); (3) decreased OXPHOS protein levels and impaired OXPHOS activities in liver, but not in other organs, (Extended Data Figs. 6c, 7a,b and 9a) consistent with an accumulation of IMT in liver (Fig. 2i); and (4) rewiring of liver metabolism towards fatty acid oxidation (Fig. 4a), concomitant with maintained fatty acid oxidation in the liver mitochondria despite markedly reduced OXPHOS capacity (Fig. 3a–d). We hypothesize that IMT treatment leads to a metabolic reprogramming in the liver that shifts the balance of dehydrogenases that deliver electrons to the Q pool (Fig. 4a,b) thereby favouring fatty acid oxidation.

The present study strongly suggests that IMT treatment increases fatty acid oxidation in the liver in mice on HFD. Future studies with isotope-labelled substrates in isolated liver cells or whole animals will be necessary to further validate this observation.

## Methods

### IMT substance and vehicle for daily gavage

The IMT substance used in this study (LDC4587) is closely related to the previously published IMT1B^6^. The IMT compound was suspended in 0.5% (*w*/*v*) hydroxypropyl methylcellulose (Hypromellose, Sigma-Aldrich, H3785) and a dose of 30 mg kg^−1^ body weight was given by gavage once per day.

### Pharmacokinetics analyses

LDC4857 was extracted from plasma and tissues by protein precipitation using acetonitrile. Tissue samples were homogenized using two parts (*w*/*v*) of PBS before extraction with acetonitrile. Following filtration, samples were analysed by liquid chromatography–tandem mass spectrometry (LC–MS/MS) using a Prominence UFLC system (Shimadzu) coupled to a Qtrap 5500 instrument (ABSciex). Test articles were separated on a C18 column using a gradient elution with an acetonitrile/water mixture containing 0.1% formic acid as the mobile phase. Chromatographic conditions and MS parameters were optimized for the tested compound before sample analysis. Concentrations of LDC4857 were calculated by means of a standard curve.

### Mouse models

C57BL/6N male mice (Charles River Laboratories) were maintained from 4 weeks of age under a 12-h light–dark cycle, temperature of 22 °C, humidity of 50% and with free access to water and standard chow diet (4% kcal from fat; Special Diet Service) or HFD (60% kcal from fat, TD.06414; Envigo). Animal studies were approved by the animal welfare ethics committee (Stockholms Djurförsöksetiska Nämnd) and performed in compliance with National and European law.

### Indirect calorimetry

The CLAMS cage system (Columbus) was used to measure food intake, O_2_ consumption, CO_2_ production, energy expenditure, RER and physical activity. Mice were individually put into metabolic cages and after 24–48 h acclimation they were visually checked for signs of stress and food/drink consumption was recorded from 9:00. If mice were not eating and/or drinking (determined as less than 1 g food or 1 ml water), they were removed from the experiment (*n* = 1 excluded mouse). From 18:00 of day three to 12:00 of day five, the data collection phase was performed. From 18:00 of day four to the 6:00 of day five, food was removed from the cage and mice were underwent a 12-h fasting period. At 6:00 of day five, food was given to the mice and data were collected for the refeeding phase. Caloric consumption was calculated using the following values: HFD 5.24 kcal g^−1^ and chow diet 3.18 kcal g^−1^.

### Energy expenditure analysis

We performed a regression-based analysis of covariance (ANCOVA)^18,19^, with total body mass as a covariate, on the energy expenditure data obtained from indirect calorimetry using the online tool CalR (https://calrapp.org).

### Lipid analysis and bomb calorimetry of faeces

For lipid extraction, a Single Mouse Metabolic Cage System (Tecniplast, 3600M021) was used for collection of mouse faeces during the fourth week of vehicle or IMT treatment. Faeces were collected for 4 days, pooled, dried overnight and lipids were extracted using Folch method^31^. For bomb calorimetry, faeces were collected at 24-h intervals over an 8-day period, weighed, dried at 63 °C for 3 days and stored at −80 °C awaiting analysis. The caloric values of faecal samples were determined using direct calorimetry in a bomb calorimeter (IKА Calorimeter System C7000) at the German Mouse Clinic. Faecal energy loss (kJ) was determined by multiplying the average daily faecal mass production by the corresponding caloric value obtained through bomb calorimetry analysis.

### BN-PAGE and OXPHOS capacity

Isolated mitochondria were analysed by BN-PAGE as previously described^32,33^. The OCRs in the liver mitochondria were assessed with high-resolution respirometry Oxygraph-2k at 37 °C. The experiments were performed by diluting 100 µg crude mitochondria diluted in 2.0 ml mitochondrial respiration medium MiR05. Oxidation of 0.2 mM malate/0.04 mM palmitoylcarnitine was monitored at state 3 with 2.5 mM ADP. To assess additive effects, the following substrates were added in addition to palmitoylcarnitine: 5 mM pyruvate/2 mM malate/10 mM glutamate, 10 mM succinate, and 10 mM glycerol-1-phosphate. To measure the succinate respiration separately, 0.5 µM rotenone/10 mM succinate was used. To measure the complex I respiration separately, two sets of substrates were used: (1) 5 mM pyruvate/2 mM malate/10 mM glutamate and (2) 2 mM malate/10 mM glutamate. Non-phosphorylating respiration (pseudo-state 4) was induced by 0.025 µM oligomycin. Mitochondrial quality was checked by 10 µM cytochrome c. The specificity of mitochondrial respiration was controlled with antimycin (0.025 µM) sensitivity. All chemicals were obtained from Sigma-Aldrich. The measurement of respiratory chain enzyme activities and citrate synthase activity was performed as previously described^32^.

### Intraperitoneal glucose tolerance test

Experiments were performed following 4 h of fasting starting at ~6:00. Blood glucose was monitored using the Contour XT glucometer (Bayer) from samples collected at the distal tail vein. Following an initial blood glucose measurement, glucose (2 g kg^−1^ body weight) was injected intraperitoneally. Blood glucose was measured 15, 30, 60, 90 and 120 min after the injection.

### Insulin secretion in isolated islets

Mouse islets were isolated as previously described^34^ and incubated in RPMI medium (11875093, Thermo Fisher Scientific) with 11 mM glucose, 10% FBS and 1% penicillin-streptomycin overnight to recover from the isolation. GSIS was performed as previously described^35^. In brief, 15 islets from each group were equilibrated in KRBH solution containing 2.8 mM glucose for 2 h, then transferred to be incubated in KRBH containing 2.8 mM glucose for 1 h, 16.7 mM glucose for another hour and the supernatant from each incubation was collected. Islets were lysed with RIPA (R0278, Sigma) containing protease inhibitor cocktail (11697498001, Roche) and phosphatase inhibitor cocktail (4906845001, Roche) to determine protein concentration. The measurement of insulin in the islet lysate, the supernatant and the fasting and 15 min ipGTT serum was performed using a Rat/Mouse Insulin ELISA kit (EZRMI-13K, Sigma-Aldrich).

### Histology and hepatic lipid quantification

One liver lobe and eWAT were collected and fixed in 4% paraformaldehyde at 4 °C for 24 h. The tissues were embedded in paraffin and sectioned to 5-μm thickness. H&E staining was performed and the morphology of the tissues was analysed by microscopy. Liver triglycerides were quantified with a kit (ab65336, Abcam) according to the manufacturer’s instructions.

### Mitochondrial isolation

Crude mitochondria from the liver were isolated by differential centrifugation in mitochondrial isolation buffer (320 mM Sucrose, 10 mM Tris-HCl, pH 7.4, 1 mM EDTA and 0.2% BSA), supplemented with EDTA‐free complete protease inhibitor cocktail and PhosSTOP Tablets (Roche). Liver tissue was homogenized using a Potter homogenizer on ice (13 strokes at 500 rpm). Nuclei and cell debris were pelleted at 1,000*g* for 10 min at 4 °C. Mitochondria were pelleted from the supernatant by centrifugation at 10,000*g* for 10 min at 4 °C. The mitochondrial pellet was carefully resuspended in mitochondrial isolation buffer without BSA and the differential centrifugations were repeated to obtain crude mitochondria.

Ultrapure mitochondria were prepared as previously described^34^. In brief, crude mitochondrial pellets from mouse liver were washed in 1xM buffer (220 mM mannitol, 70 mM sucrose, 5 mM HEPES, pH 7.4 and 1 mM EGTA, pH 7.4); pH was adjusted with potassium hydroxide, supplemented with EDTA‐free complete protease inhibitor cocktail and PhosSTOP Tablets (Roche) and purified on a Percoll density gradient (12%:19%:40%) via centrifugation in a SW41 rotor at 42,000*g* at 4 °C for 1 h in a Beckman Coulter Optima L‐100 XP ultracentrifuge using 14 × 89-mm Ultra‐Clear Centrifuge Tubes (Beckman Instruments). Mitochondria were collected at the interphase between 19 and 40% Percoll and washed three times with buffer 1xM. The mitochondrial pellets were then frozen at −80 °C.

### Western blots

Mitochondrial proteins (10 μg) were resuspended in 1× NuPAGE LDS sample buffer. Mitochondrial proteins were thereafter separated by SDS–PAGE (4–12% Bis‐Tris Protein Gels; Invitrogen) and transferred onto polyvinylidene difluoride membranes (Merck Millipore). Immunoblotting was performed using standard procedures with ECL reagent detection as previously decribed^32^.

### RNA extraction and RT–qPCR

RNA was extracted from mouse liver, skeletal muscle, eWAT, heart and BAT using Trizol reagent (Invitrogen) according to the manufacturer’s instructions and then treated with TURBO DNA‐free DNase (Invitrogen). For RT–qPCR expression analysis, complementary DNA was reversed transcribed from 1 μg total RNA using the High‐Capacity cDNA Reverse Transcription kit (Invitrogen). The qPCR was performed in a QuantStudio 6 Flex Real‐Time PCR System (Life Technologies), using TaqMan Universal Master Mix II with UNG (Applied Biosystems) to quantify mitochondrial transcripts (mt‐rRNAs and mt‐mRNAs), actin and 18S rRNA.

### DNA isolation and mtDNA quantification

Genomic DNA was isolated from mouse liver, eWAT and skeletal muscle using the DNeasy Blood and Tissue kit (QIAGEN) following the manufacturer’s instructions and treated with RNase A. Levels of mtDNA were measured by quantitative PCR using 5 ng DNA in a QuantStudio 6 Flex Real‐Time PCR System using TaqMan Universal Master Mix II with UNG. Nd1, Cox1 and Cyb probes were used for TaqMan assays to measure mtDNA levels and 18S was used for normalization.

### Label‐free quantitative proteomics

#### Proteomics sample preparation

Frozen tissue pieces were placed in precooled ‘Lysing Matrix D’ tubes, followed by addition of 400 µl lysis buffer (1% SDC in 100 mM Tris-HCl, pH 8.5). Tissue pieces were lysed at 4 °C by three cycles of 40 s bead beating (6.0 setting) and 20 s pause in the FastPrep-24 (MP Biomedicals). Thereafter, lysates were transferred into reaction tubes and boiled for 10 min at 95 °C. Similarly, ultrapure mitochondria pellets were resuspended in 150 µl lysis buffer and boiled for 10 min at 95 °C. After lysate boiling, the protein concentration was estimated by tryptophan assay and 30 µg of each sample were diluted with lysis buffer to a protein concentration of 0.75 µg µl^−1^. Proteins were reduced and alkylated by adding chloroacetamide (CAA) and Tris(2-carboxyethyl)phosphine (TCEP) to a final concentration of 40 mM and 10 mM, respectively in a 5-min incubation at 45 °C. After adding trypsin (1:100 (*w*/*w*), Sigma-Aldrich) and LysC (1:100 (*w*/*w*), Wako), proteins were digested overnight at 37 °C. Protein digestion was quenched by adding 200 µl 1% TFA in isopropanol to the samples. Subsequently, peptides were loaded onto SDB-RPS StageTips (Empore) followed by washes with 200 µl 1% TFA in isopropanol and 200 µl 0.2% TFA in 2% ACN. Peptides were eluted with 60 µl 1.25% NH_4_OH in 80% ACN and dried in a SpeedVac centrifuge (Eppendorf, Concentrator Plus). Dried peptides were resuspended in A* (0.2% TFA in 2% ACN) and subjected to measurement by LC–MS/MS.

#### LC–MS/MS and proteomics data analysis

Peptide concentration was estimated by NanoDrop and 250 ng peptide material was used for individual measurements. Peptides were loaded onto a 50-cm, in-house packed, reversed-phase column (75-μm inner diameter, ReproSil-Pur C18-AQ 1.9 μm resin, Dr. Maisch) and separated with a binary buffer system consisting of buffer A (0.1% FA) and buffer B (0.1% FA in 80% ACN) with an EASY-NLC 1,200 (Thermo Fisher Scientific). The LC system was directly coupled online with the mass spectrometer (Exploris 480, Thermo Fisher Scientific) via a nano-electrospray source. Peptide separation was performed at a flow rate of 300 µl min^−1^ and an elution gradient starting at 5% B increasing to 30% B in 80 min, 60% in 4 min and 95% in 4 min.

Data were acquired in DIA mode with a scan range of 300–1,650 *m*/*z* at a resolution of 120,000. The AGC was set to 3 × 10^6^ at a maximum injection time of 60 ms. Precursor fragmentation was achieved via HCD (NCD 25.5%, 27.5% and 30%) and fragment ions were analysed in 33 DIA windows at a resolution of 30,000, while the AGC was kept at 1 × 10^6^.

DIA raw files were processed using Spectronaut (v.14) with default settings. Perseus (v.1.6.7.0)^36^ was used on data with three valid values in at least one treatment group. PCA was performed on missing-values imputed matrices and analysis of variance (ANOVA) testing with permutation-based FDR correction. GSEA was computed with WebGestalt 2019 (ref. ^37^) in an R environment (v.4.1.2) correcting for multiple library testing and normalized enrichment scores were reported. Statistical analyses and FDR calculations were performed with limma and an FDR cutoff of 0.05 was defined as significant. Heatmaps were generated on filtered, imputed and *z*-transformed data matrices in R with the pheatmap package.

### Metabolomics and lipidomics

#### Samples extraction of polar and lipid metabolites

Metabolites were extracted from 10–15 mg of frozen tissue. The frozen tissue samples were homogenized to a fine tissue powder using a ball mill (MM400, Retsch).

After the tissue was pulverized in 2-ml round-bottom microcentrifuge tubes, a 1-ml −20 °C methyl-tert butyl-ether:methanol:water (5:3:2 (*v*/*v*/*v*)) mixture, containing 0.2 µl ml^−1^ deuterated EquiSplash lipidomix (Avanti), 0.2 µl ml^−1^ U-^13^C^15^N amino acid mix (Cambridge Isotopes, MSK_A2-1.2), 0.1 μl ml^−1^ 1 mg ml^−1^ ^13^C_10_ ATP, ^15^N_5_ ADP and ^13^C_10_^15^N_5_ AMP (Sigma) and 0.2 µl ml^−1^ 100 µg ml^−1^ of deuterated citric acid as internal standards was added to each sample. After addition of the extraction buffer, the samples were immediately vortexed before they were incubated for additional 30 min at 4 °C on an orbital shaker. Proteins were removed by a 10-min 21,000*g* centrifugation at 4 °C and the supernatant was transferred to a fresh 2-ml Eppendorf tube. To separate the organic from the polar phase, 150 μl MTBE and 100 µl UPC/MS-grade water was added to the cleared supernatant, which was briefly vortexed before mixing it for 15 min at 15 °C on an orbital shaker. Phase separation was obtained after a 5 min centrifugation at 16,000*g* at 15 °C. The upper MTBE phase, which contains the hydrophobic compounds (lipids), was sampled to a fresh 1.5-ml microcentrifuge tube (~600 µl), while the remaining polar phase (~600 µl) was kept in the initial 2-ml tube. These two fractions were then immediately concentrated to dryness in a speed vacuum concentrator (LaboGene, MaxiVac) at room temperature. Thereafter, samples were then either stored at −80 °C or processed immediately for LC–MS analysis.

#### LC–high-resolution MS-based analysis of anionic and amine-containing metabolites from the polar fraction

The polar fraction of the extracted metabolites was resuspended in 400 µl ULC–MS-grade water (Biosolve). After 15 min of incubation on a thermomixer at 4 °C and a 5-min centrifugation at 16,000*g* at 4 °C, 100 µl of the cleared supernatant were transferred to polypropylene autosampler vials (Chromatography Accessories Trott) and analysed using anion-exchange chromatography MS (AEX-MS), described in detail previously^6,38^. For the analysis of amine-containing compounds, 50 µl of the above-mentioned resuspended 400 µl polar phase were mixed with 25 µl 100 mM sodium carbonate (Sigma), followed by the addition of 25 µl 2% (*v*/*v*) benzoylchloride (Sigma) in acetonitrile (UPC–MS-grade, Biosolve). Derivatized samples were thoroughly mixed and analysed as previously described^6,38^.

#### LC–high-resolution MS-based analysis of lipids

The dried lipid fractions were resuspended in 400 µl UPLC-grade acetonitrile: isopropanol (70:30 (*v*/*v*), Biosolve). Samples were vortexed for 10 s and incubated for 10 min on a thermomixer at 4 °C. Resuspended samples were centrifuged for 5 min at 10,000*g* and 4 °C, before transferring the cleared supernatant to 2-ml glass vials with 200-µl glass inserts (Chromatography Zubehör Trott). All samples were placed in a UHPLC sample manager (Vanquish, Thermo Fisher Scientific), which was set to 6 °C. The UHPLC was connected to a Tribrid Orbitrap HRMS, equipped with a heated ESI source (ID-X, Thermo Fisher Scientific).

A volume of 1 µl of each lipid sample was injected into a 100 × 2.1-mm BEH C8 UPLC column, packed with 1.7-µm particles (Waters). The flow rate of the UPLC was set to 400 µl min^−1^ and the buffer system consisted of buffer A (10 mM ammonium acetate, 0.1% acetic acid in UPLC-grade water) and buffer B (10 mM ammonium acetate, 0.1% acetic acid in UPLC-grade acetonitrile/isopropanol 7:3 (*v*/*v*)). The UPLC gradient was as follows: 0–1 min in 45% A, 1–4 min in 45–25% A, 4–12 min in 25–11% A, 12–15 min in 11–1% A, 15–20 min in 1% A, 20–20.1 min in 1–45% A and 20.1–24 min re-equilibrating at 45% A, which leads to a total runtime of 24 min per sample and polarity.

The ID-X mass spectrometer was operating for the first injection in positive ionization mode and for the second injection in negative ionization mode. In both cases, the analysed mass range was between *m*/*z* 150–1,500. The resolution was set to 120,000, leading to approximately four scans per second. The RF lens was set to 50% and the AGC target was set to 100%. The maximal ion time was set to 100 ms and the heated ESI source was operating with a spray voltage of 3.6 kV in positive ionization mode, while 3.2 kV was applied in negative ionization mode. The ion tube transfer capillary temperature was 300 °C, the sheath gas flow was 60 arbitrary units (AU), the auxiliary gas flow was 20 AU and the sweep gas flow was set to 1 AU at 330 °C.

To obtain positive ionization mode and negative ionization mode MS/MS-based lipid annotations, we performed five iterative MS/MS deep-sequencing runs on a pooled sample of each tissue type using the AcquireX algorithm (Xcalibur v.4.3, Thermo Fisher Scientific). Lipids from these MS/MS spectra were then automatically annotated using LipidSearch (v.4.2, Thermo Fisher Scientific). The annotated lipids were filtered for quality grades (A,B and C were accepted) and the resulting lipid IDs, *m*/*z* and retention time values were exported into a TraceFinder compound database (v.4.1, Thermo Fisher Scientific). From this compound database we generated a TraceFinder method for each sample set and extracted the corresponding peaks from each full-scan MS spectrum.

For data analysis, the area of each monoisotopic mass peak was extracted and integrated using a mass accuracy of <5 ppm and a retention time tolerance of <0.05 min compared with the independently measured reference compounds. Areas of the cellular pool sizes were normalized to the internal standards, followed by a normalization to the fresh weight/volume of the analysed sample.

### Statistical analysis

Experiments were replicated across multiple batches. Within each batch, mice were randomized into groups and treated according to the experimental design. This approach ensured that the results were not dependent on a single cohort and increased the generalizability and confidence in our findings. Due to body size differences, complete blindness during data collection and analysis was challenging for HFD-fed mice. Biochemical experiments were independently performed at least three times and results represent *n* > 5 independent biological replicates, unless indicated otherwise. No data points were excluded. All values are presented as mean ± s.e.m. Statistical analyses were conducted using GraphPad Prism software (v.9.4.0). Before statistical analysis, data were tested for normal distribution using the Kolmogorov–Smirnov test with Lillifors correction or D’Agostino–Pearson omnibus test. Statistical significance was assessed by a two-way ANOVA with Tukey’s test for multiple comparisons in normal distributed data. For non-normal distribution, data were analysed using a Mann–Whitney *U*-test, as indicated in the figure legends. *P* values are shown in the figures. No statistical methods were used to predetermine sample sizes but our sample sizes are similar to those reported in previous publications^32,39^.

### Reporting summary

Further information on research design is available in the Nature Portfolio Reporting Summary linked to this article.

### Supplementary information

Reporting Summary

Supplementary Table 1 Table with TaqMan probes used in manuscript.

### Source data

Source Data Fig. 1 Statistical source data.

Source Data Fig. 2 Statistical source data.

Source Data Fig. 3 Statistical source data.

Source Data Extended Data Fig. 1 Statistical source data.

Source Data Extended Data Fig. 2 Statistical source data.

Source Data Extended Data Fig. 3 Statistical source data.

Source Data Extended Data Fig. 4 Statistical source data.

Source Data Extended Data Fig. 5 Statistical source data.

Source Data Extended Data Fig. 5 Unprocessed western blots.

Source Data Extended Data Fig. 6 Statistical source data.

Source Data Extended Data Fig. 7 Unprocessed western blots.

Source Data Extended Data Fig. 7 Statistical source data.

Source Data Extended Data Fig. 8 Statistical source data.

Source Data Extended Data Fig. 9 Statistical source data.

Source Data Extended Data Fig. 10 Statistical source data.

Source Data Extended Data Fig. 10 Unprocessed western blots.

## Data Availability

The mass spectrometry proteomics data have been deposited to the ProteomeXchange Consortium via the PRIDE partner repository^40^ with the dataset identifier PXD034771. All data and materials used in this study, including standard code with no custom code generated, are available in Source Data with no restrictions. Source data are provided with this paper.
