# Supplementary material for: Inhibition of mammalian mtDNA transcription acts paradoxically to reverse diet-induced hepatosteatosis and obesity
Source: Nat Metab. 2024 Apr 30;6(6):1024–35. doi: 10.1038/s42255-024-01038-3 (PMC11199148; doi:10.1038/s42255-024-01038-3)
Supplement: Supplementary file 1 — Reporting Summary [file 42255_2024_1038_MOESM1_ESM.pdf]

nature portfolio

Corresponding author(s): Nils-Göran Larsson

Last updated by author(s): Mar 14, 2024

Reporting Summary

Nature Portfolio wishes to improve the reproducibility of the work that we publish. This form provides structure for consistency and transparency in reporting. For further information on Nature Portfolio policies, see our Editorial Policies and the Editorial Policy Checklist.

Statistics

For all statistical analyses, confirm that the following items are present in the figure legend, table legend, main text, or Methods section.

n/a	Confirmed	
☐	☒	The exact sample size (*n*) for each experimental group/condition, given as a discrete number and unit of measurement
☐	☒	A statement on whether measurements were taken from distinct samples or whether the same sample was measured repeatedly
☐	☒	The statistical test(s) used AND whether they are one- or two-sided<br>*Only common tests should be described solely by name; describe more complex techniques in the Methods section.*
☐	☒	A description of all covariates tested
☐	☒	A description of any assumptions or corrections, such as tests of normality and adjustment for multiple comparisons
☐	☒	A full description of the statistical parameters including central tendency (e.g. means) or other basic estimates (e.g. regression coefficient) AND variation (e.g. standard deviation) or associated estimates of uncertainty (e.g. confidence intervals)
☐	☒	For null hypothesis testing, the test statistic (e.g. *F*, *t*, *r*) with confidence intervals, effect sizes, degrees of freedom and *P* value noted<br>*Give P values as exact values whenever suitable.*
☒	☐	For Bayesian analysis, information on the choice of priors and Markov chain Monte Carlo settings
☒	☐	For hierarchical and complex designs, identification of the appropriate level for tests and full reporting of outcomes
☒	☐	Estimates of effect sizes (e.g. Cohen's *d*, Pearson's *r*), indicating how they were calculated

Our web collection on statistics for biologists contains articles on many of the points above.

Software and code

Policy information about availability of computer code

Data collection	Oroboros Datlab 7.4 was used for Oroboros experiments.<br>AcquireX algorithm (Xcalibur Version 4.3, Thermo Fisher Scientific) was used to obtain MS/MS-based lipid annotations.<br>ImageLab 6.1 was used for visulize the western exposure and annotating molecular weight marker.<br>QuantStudio 6 Flex Real-Time PCR Software v1.3 (applied biosystems) was used for collecting data for qPCR.<br>Gen5 1.05 was used for colorimetric assays measuring including liver triglycerides, serum ALT, serum AST, serum albumin, serum and islets insulin content.<br>Oxymax/CLAMS Software Version 5.40 was used to collect metabolic data from Oxymax/Comprehensive Lab Animal Monitoring System.
Data analysis	CalR (v1.3) (https://calrapp.org/) for energy expenditure, web-tool.<br>Gene Set Enrichment Analysis were computed with WebGestalt 201959 in an R environment (v4.1.2), web-tool.<br>GraphPad Prism software (v.9.4.0) was used for data analysis, making plots and statistical analyses.<br>Gene Set Enrichment Analyses were computed with WebGestalt 2019 (web application) in R environment (v4.1.2).<br>Spectronaut (version 14, Biognosys) was used to process DIA raw files from proteomics.<br>Perseus (v1.6.7.0) was used for proteomics data processing and initial data analysis.<br>LipidSearch (version 4.2, Thermo Fisher Scientific) was used for identifying and annotating lipids from MS/MS spectra.<br>TraceFinder software (Version 4.1, Thermo Fisher Scientific) was used for semi-targeted LC-MS data analysis and lipid data analysis.

For manuscripts utilizing custom algorithms or software that are central to the research but not yet described in published literature, software must be made available to editors and reviewers. We strongly encourage code deposition in a community repository (e.g. GitHub). See the Nature Portfolio guidelines for submitting code & software for further information.

Data

Policy information about availability of data

All manuscripts must include a data availability statement. This statement should provide the following information, where applicable:

- Accession codes, unique identifiers, or web links for publicly available datasets
- A description of any restrictions on data availability
- For clinical datasets or third party data, please ensure that the statement adheres to our policy

The mass spectrometry proteomics raw data have been deposited to the ProteomeXchange Consortium via the PRIDE partner repository with the dataset identifier PXD034771. All data and materials used in this study, including standard code with no custom code generated, are available in Source Data with no restrictions.

Research involving human participants, their data, or biological material

Policy information about studies with human participants or human data. See also policy information about sex, gender (identity/presentation), and sexual orientation and race, ethnicity and racism.

Reporting on sex and gender	N/A
Reporting on race, ethnicity, or other socially relevant groupings	N/A
Population characteristics	N/A
Recruitment	N/A
Ethics oversight	N/A

Note that full information on the approval of the study protocol must also be provided in the manuscript.

Field-specific reporting

Please select the one below that is the best fit for your research. If you are not sure, read the appropriate sections before making your selection.

☒ Life sciences ☐ Behavioural & social sciences ☐ Ecological, evolutionary & environmental sciences

For a reference copy of the document with all sections, see nature.com/documents/nr-reporting-summary-flat.pdf

Life sciences study design

All studies must disclose on these points even when the disclosure is negative.

Sample size	Sample sizes for each experimental group were determined based on a combination of factors, including previous literature, ethical permits, and practical constraints. While no formal sample size calculations were performed, we followed a common approach used in research studies involving mice, where sample sizes are often based on historical data and empirical observations 1. Our decision considered the variability observed in similar interventions, anticipated effect size from preliminary data, and known physiological responses. Ethical principles guided us to minimize animal usage while ensuring statistical robustness. We aimed to balance achieving statistical power with practical feasibility given the inherent variability in mouse models and the multifactorial nature of obesity 2.<br>References:<br>1 Festing, M. F. & Altman, D. G. Guidelines for the design and statistical analysis of experiments using laboratory animals. ILAR J 43, 244-258 (2002). https://doi.org/10.1093/ilar.43.4.244<br>2 Charan, J. & Kantharia, N. D. How to calculate sample size in animal studies? J Pharmacol Pharmaco 4, 303-306 (2013). https://doi.org/Pmid 24250214<br>10.4103/0976-500x.119726
Data exclusions	One mouse was excluded from metabolic cage study, because of sign of inadaptation to metabolic cage (eat less than 1 gram of food or drink less than 1 ml of water).
Replication	All mouse experiments were replicated across multiple batches, with each batch consisting of independent cohorts of mice subjected to the same experimental conditions. Specifically, each experimental condition. Within each batch, mice were randomized into groups and treated according to the experimental design. This approach ensured that the results were not dependent on a single cohort of animals and increased the generalizability and confidence in our findings.<br>All the biochemical experiments were performed independently for at least three times, and results are representative of n > 5 independent biological replicates, unless indicated otherwise.
Randomization	Mice were randomized for treatment and diets.

Blinding	Mice were randomized to different groups but blinding was not possible as effects of diets were clearly visible from body size.

Reporting for specific materials, systems and methods

We require information from authors about some types of materials, experimental systems and methods used in many studies. Here, indicate whether each material, system or method listed is relevant to your study. If you are not sure if a list item applies to your research, read the appropriate section before selecting a response.

Materials & experimental systems

n/a	Involved in the study	
☐	☒	Antibodies
☒	☐	Eukaryotic cell lines
☒	☐	Palaeontology and archaeology
☐	☒	Animals and other organisms
☒	☐	Clinical data
☒	☐	Dual use research of concern
☒	☐	Plants

Methods

n/a	Involved in the study	
☒	☐	ChIP-seq
☒	☐	Flow cytometry
☒	☐	MRI-based neuroimaging

Antibodies

Antibodies used	Total OXPHOS Rodent WB Antibody Cocktail (Abcam, ab110413, dilution 1:1000), contains 5 mouse mAbs, one each against CI subunit NDUFB8 (ab110242), CII subunit SDHB (ab14714), CIII subunit UQCRC2 (ab14745), CIV subunit MTCO1 (ab14705) and CV subunit ATP5A (ab14748).<br>VDAC (Abcam, ab14734, dilution 1:1000)<br>ACC (Abcam, ab45174, dilution 1:1000)<br>phosACC-Ser79 (Abcam, ab68191, dilution 1:500)<br>SDHA (Abcam, ab14715, dilution 1:2000)<br>AMPKα (Cell Signaling Technology #2532, dilution 1:1000)<br>phosAMPKα-Thr172 (Cell Signaling Technology #2535, dilution 1:1000)
Validation	Antibodies include OXPHOS, VDAC, SDHA, AMPKα, phosAMPKα-Thr172 were previously validated in our lab, e.g., Jiang, S. et al and Bonekamp, N. A. et al.<br>Jiang, S. et al. TEFM regulates both transcription elongation and RNA processing in mitochondria. EMBO Rep 20 (2019). https://doi.org:10.15252/embr.201948101<br>Bonekamp, N. A. et al. Small-molecule inhibitors of human mitochondrial DNA transcription. Nature 588, 712-716 (2020). https://doi.org:10.1038/s41586-020-03048-z<br><br>Antibodies include VDAC, SDHA, ACC and phosACC-Ser79 validations were provided on the manufacturer's website, for both are "knockout validated".

Animals and other research organisms

Policy information about studies involving animals; ARRIVE guidelines recommended for reporting animal research, and Sex and Gender in Research

Laboratory animals	M. musculus, C57BL/6N. only males, age 4-16 weeks. housing condition: 22°C, 50% humidity.
Wild animals	No wild animals were used in this study.
Reporting on sex	Only male mice were used. Less variation as estrous cycle is not confounding results.
Field-collected samples	No field collected samples were used in this study.
Ethics oversight	Stockholms djurförsöksetiska nämnd

Note that full information on the approval of the study protocol must also be provided in the manuscript.

Plants

Seed stocks	*Report on the source of all seed stocks or other plant material used. If applicable, state the seed stock centre and catalogue number. If plant specimens were collected from the field, describe the collection location, date and sampling procedures.*
Novel plant genotypes	*Describe the methods by which all novel plant genotypes were produced. This includes those generated by transgenic approaches, gene editing, chemical/radiation-based mutagenesis and hybridization. For transgenic lines, describe the transformation method, the number of independent lines analyzed and the generation upon which experiments were performed. For gene-edited lines, describe the editor used, the endogenous sequence targeted for editing, the targeting guide RNA sequence (if applicable) and how the editor was applied.*
Authentication	*Describe any authentication procedures for each seed stock used or novel genotype generated. Describe any experiments used to assess the effect of a mutation and, where applicable, how potential secondary effects (e.g. second site T-DNA insertions, mosiacism, off-target gene editing) were examined.*
