# Supplementary figures and images for: Inhibition of mammalian mtDNA transcription acts paradoxically to reverse diet-induced hepatosteatosis and obesity
Source: Nat Metab. 2024 Apr 30;6(6):1024–35. doi: 10.1038/s42255-024-01038-3 (PMC11199148; doi:10.1038/s42255-024-01038-3)

Western gel for Extended Data Fig.5d

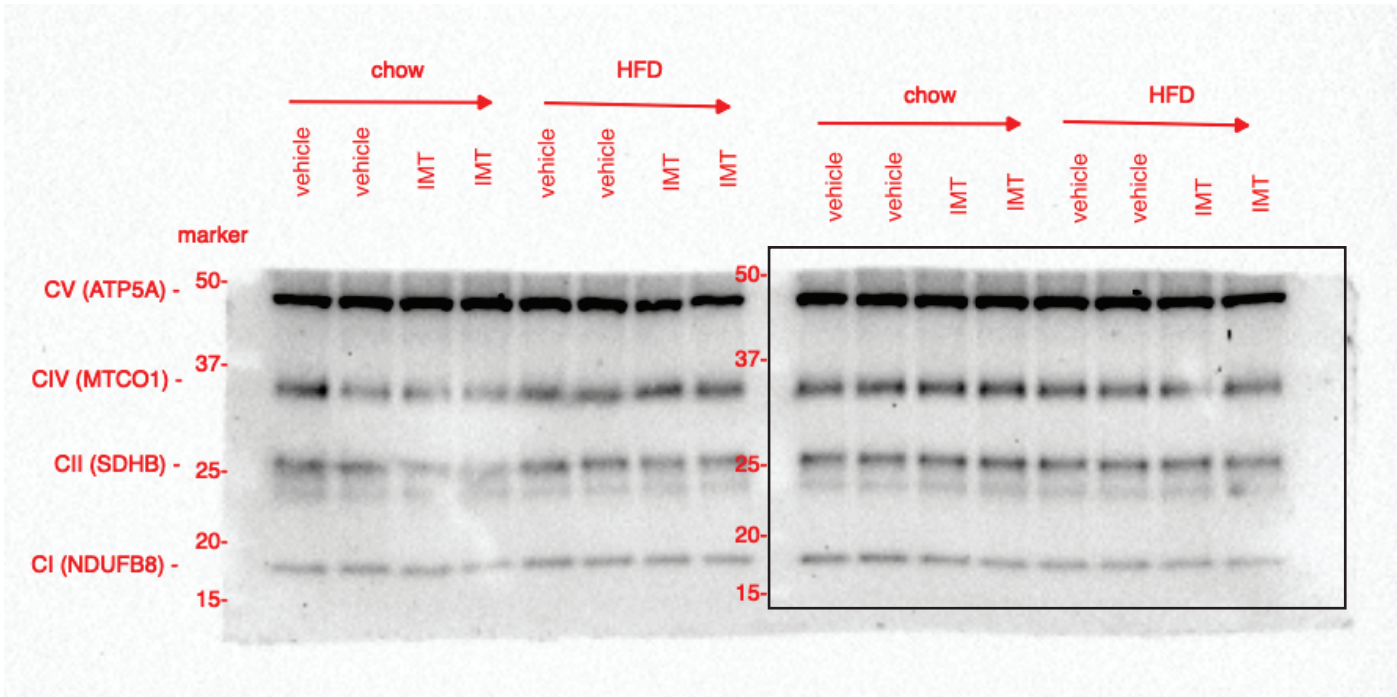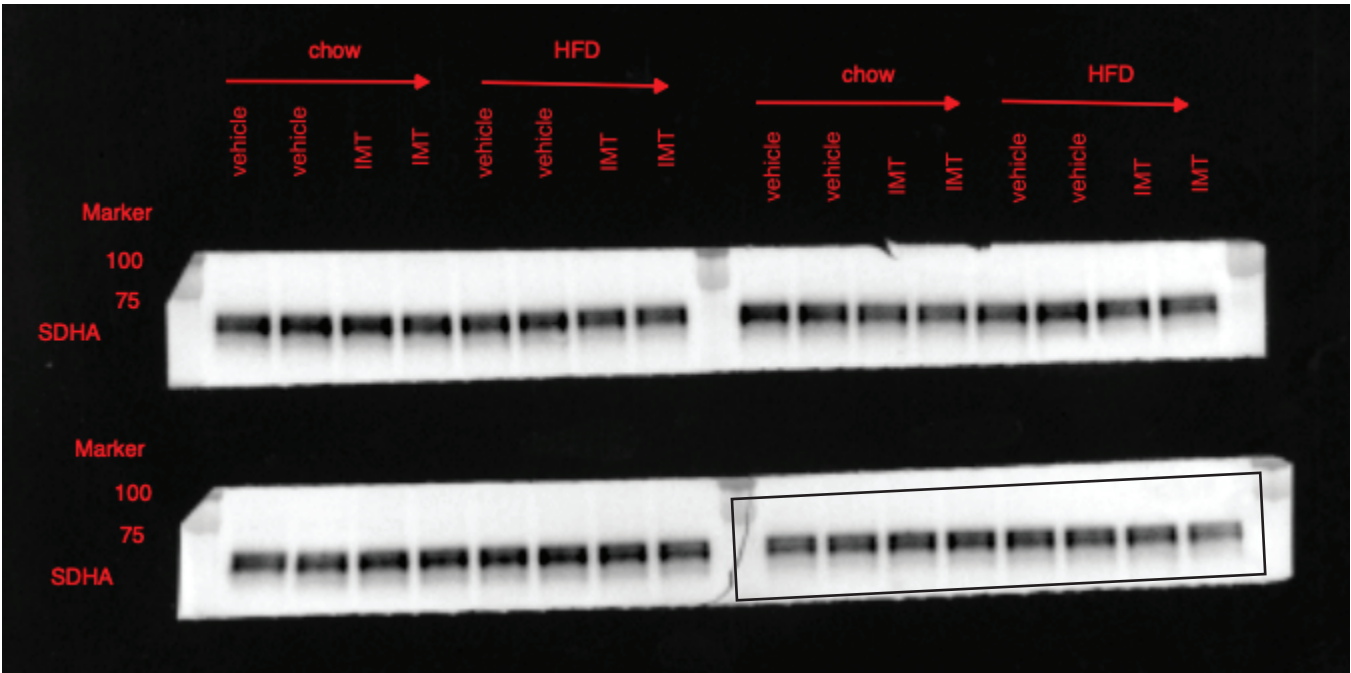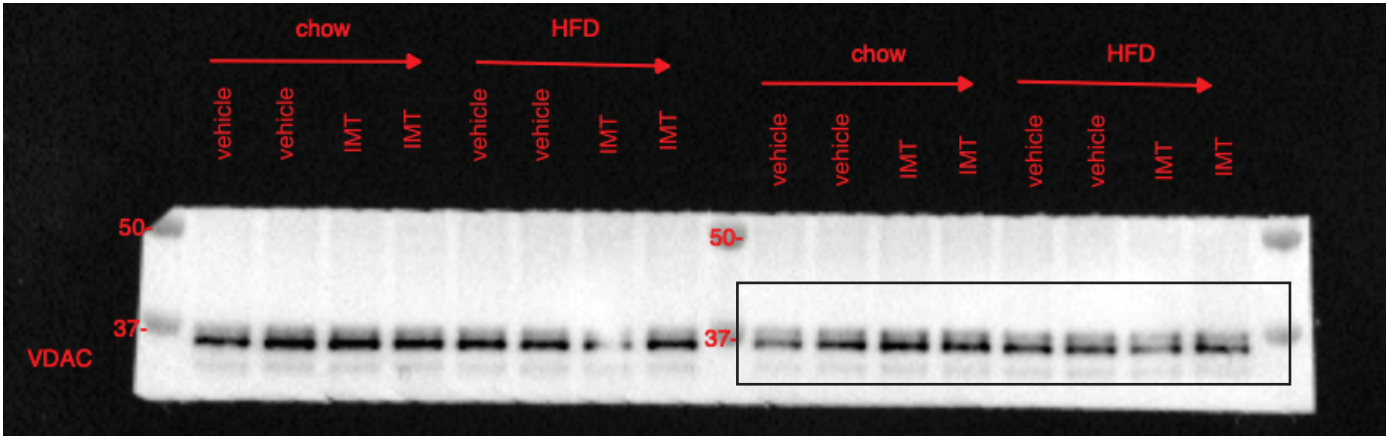

Supplement: Supplementary file 11 — Unprocessed western blots. [file 42255_2024_1038_MOESM11_ESM.pdf]

Western gel for Extended Data Fig. 7b

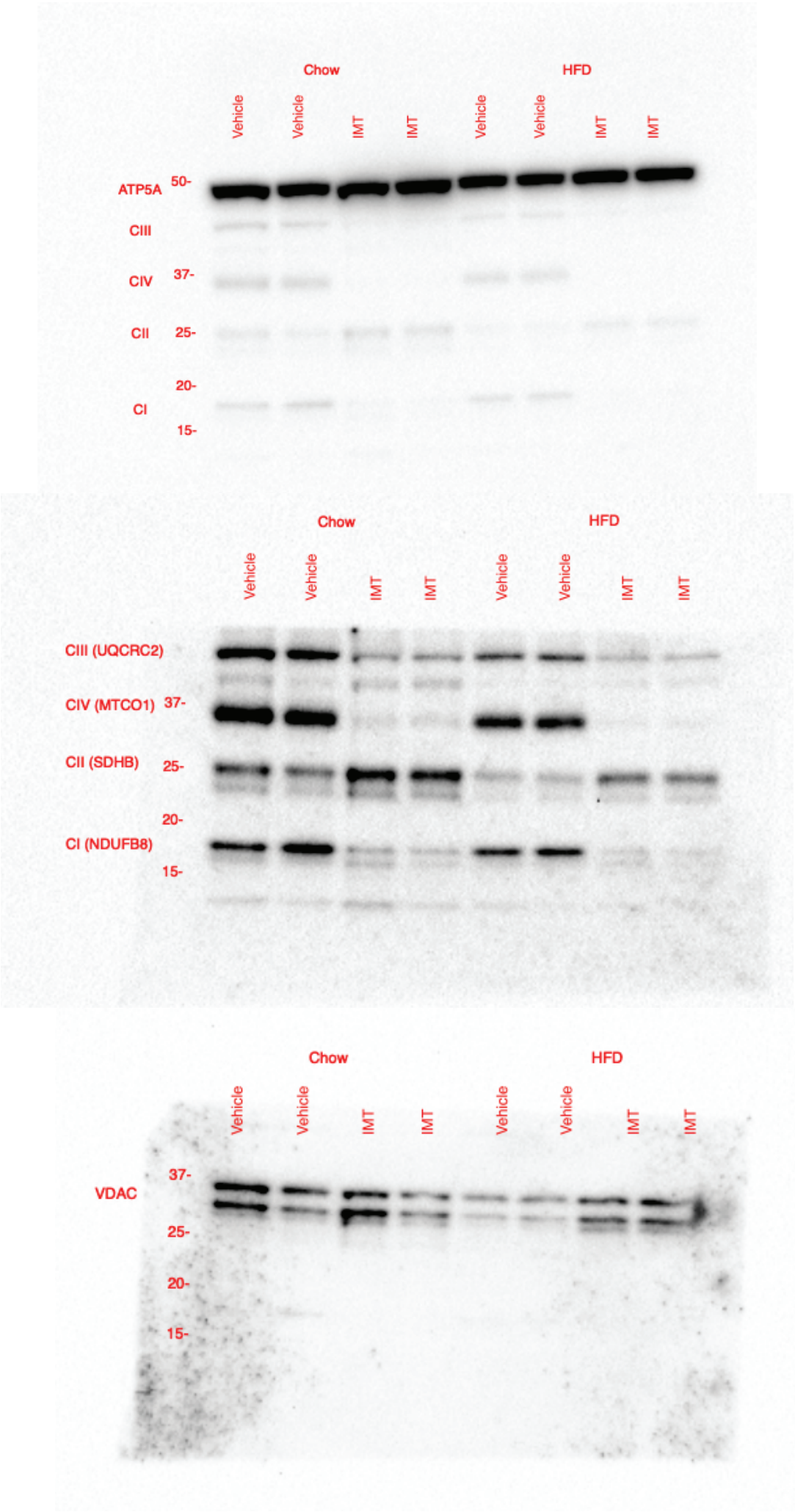

Supplement: Supplementary file 13 — Unprocessed western blots. [file 42255_2024_1038_MOESM13_ESM.pdf]

Western gel for Extended Data Fig. 10g

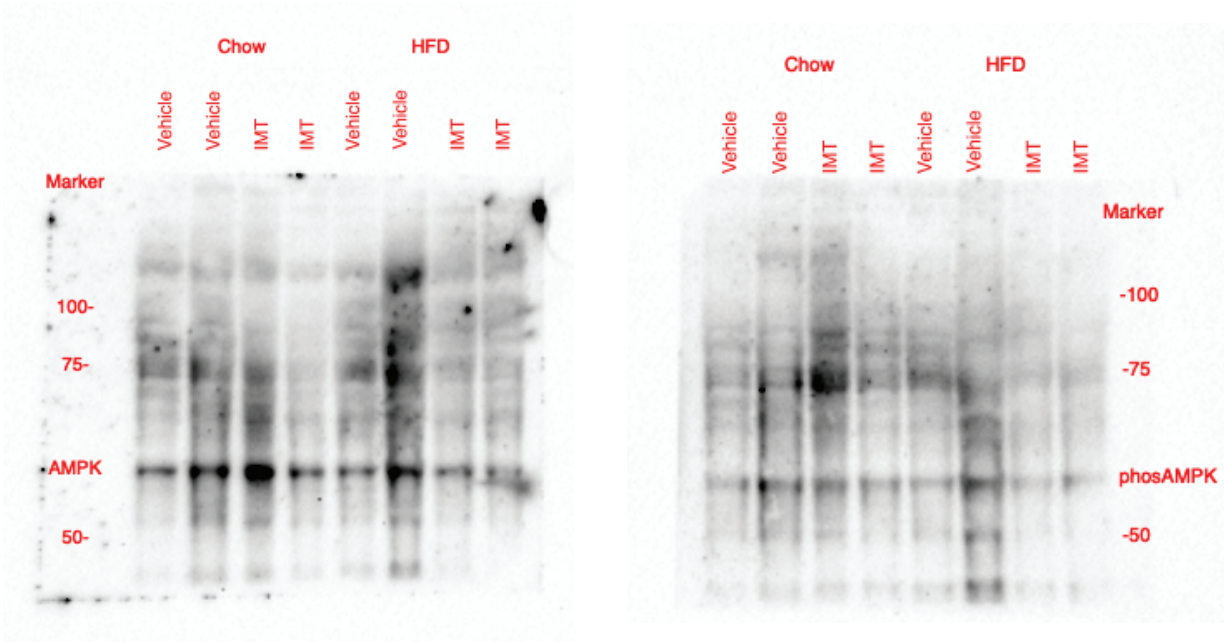

Short exposure

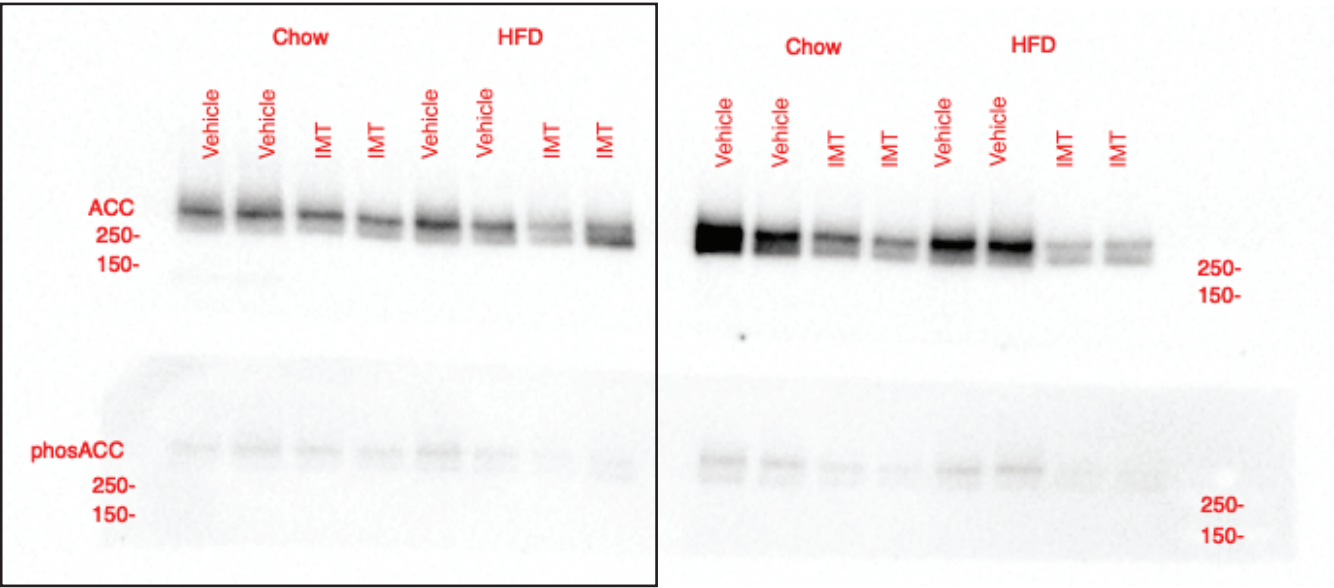

Long exposure

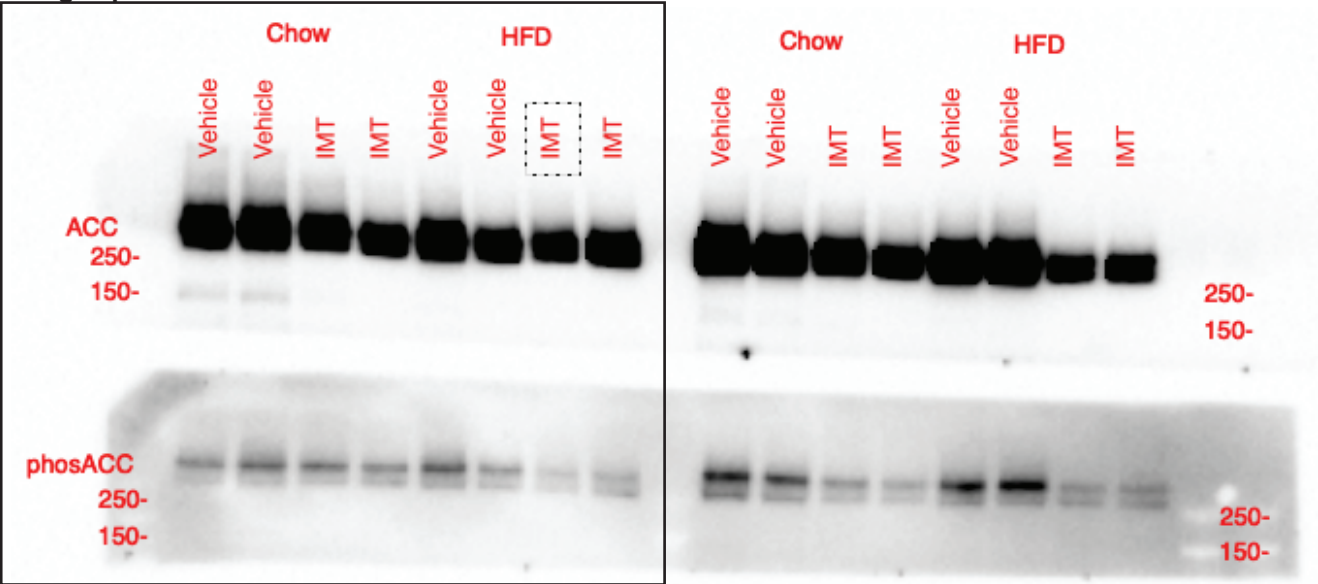

Supplement: Supplementary file 18 — Unprocessed western blots. [file 42255_2024_1038_MOESM18_ESM.pdf]
